# Early white matter development is abnormal in tuberous sclerosis complex patients who develop autism spectrum disorder

**DOI:** 10.1186/s11689-019-9293-x

**Published:** 2019-12-16

**Authors:** Anna K. Prohl, Benoit Scherrer, Xavier Tomas-Fernandez, Peter E. Davis, Rajna Filip-Dhima, Sanjay P. Prabhu, Jurriaan M. Peters, E. Martina Bebin, Darcy A. Krueger, Hope Northrup, Joyce Y. Wu, Mustafa Sahin, Simon K. Warfield, on behalf of the TACERN Study Group, Simon K. Warfield, Jurriaan M. Peters, Monisha Goyal, Deborah A. Pearson, Marian E. Williams, Ellen Hanson, Nicole Bing, Bridget Kent, Sarah O’Kelley, Rajna Filip-Dhima, Kira Dies, Stephanie Bruns, Benoit Scherrer, Gary Cutter, Donna S. Murray, Steven L. Roberds

**Affiliations:** 1 Computational Radiology Laboratory, Department of Radiology, Boston Children’s Hospital, Harvard Medical School, Harvard University, Boston, Massachusetts USA; 2 Department of Neurology, Boston Children’s Hospital, Harvard Medical School, Harvard University, Boston, Massachusetts USA; 3 Department of Neurology, University of Alabama at Birmingham, Birmingham, Alabama USA; 4 Department of Neurology and Rehabilitation Medicine, Cincinnati Children’s Hospital Medical Center, Cincinnati, Ohio USA; 5 Department of Pediatrics, McGovern Medical School, University of Texas Health Science Center at Houston, Houston, Texas USA; 6 Division of Pediatric Neurology, University of California at Los Angeles Mattel Children’s Hospital, David Geffen School of Medicine, University of California, California, Los Angeles USA; 7 F.M. Kirby Neurobiology Center, Boston Children’s Hospital, Harvard Medical School, Harvard University, Boston, Massachusetts USA

**Keywords:** Autism spectrum disorder, Tuberous sclerosis complex, Infant brain development, Diffusion tensor imaging, White matter

## Abstract

**Background:**

Autism spectrum disorder (ASD) is prevalent in tuberous sclerosis complex (TSC), occurring in approximately 50% of patients, and is hypothesized to be caused by disruption of neural circuits early in life. Tubers, or benign hamartomas distributed stochastically throughout the brain, are the most conspicuous of TSC neuropathology, but have not been consistently associated with ASD. Widespread neuropathology of the white matter, including deficits in myelination, neuronal migration, and axon formation, exist and may underlie ASD in TSC. We sought to identify the neural circuits associated with ASD in TSC by identifying white matter microstructural deficits in a prospectively recruited, longitudinally studied cohort of TSC infants.

**Methods:**

TSC infants were recruited within their first year of life and longitudinally imaged at time of recruitment, 12 months of age, and at 24 months of age. Autism was diagnosed at 24 months of age with the ADOS-2. There were 108 subjects (62 TSC-ASD, 55% male; 46 TSC+ASD, 52% male) with at least one MRI and a 24-month ADOS, for a total of 187 MRI scans analyzed (109 TSC-ASD; 78 TSC+ASD). Diffusion tensor imaging properties of multiple white matter fiber bundles were sampled using a region of interest approach. Linear mixed effects modeling was performed to test the hypothesis that infants who develop ASD exhibit poor white matter microstructural integrity over the first 2 years of life compared to those who do not develop ASD.

**Results:**

Subjects with TSC and ASD exhibited reduced fractional anisotropy in 9 of 17 white matter regions, sampled from the arcuate fasciculus, cingulum, corpus callosum, anterior limbs of the internal capsule, and the sagittal stratum, over the first 2 years of life compared to TSC subjects without ASD. Mean diffusivity trajectories did not differ between groups.

**Conclusions:**

Underconnectivity across multiple white matter fiber bundles develops over the first 2 years of life in subjects with TSC and ASD. Future studies examining brain-behavior relationships are needed to determine how variation in the brain structure is associated with ASD symptoms.

## Background

Autism spectrum disorders (ASDs) are a group of genetically and phenotypically heterogeneous neurodevelopmental disorders unified by impairment in social interaction and communication and the presence of repetitive, stereotypic behaviors [1]. These behaviors present within the first 2 years of life and affect 1–2% of children worldwide [2]. Although prevalent, genetic and phenotypic complexity has limited the field’s understanding and treatment of ASDs. Hundreds of genetic variants acquired through multiple inheritance patterns and forms of genetic mutation have been associated with ASDs, and expression and severity of the core symptoms are heterogeneous. There is also a diversity of neuropsychiatric and somatic conditions comorbid with ASDs, such as intellectual disability, epilepsy, attention-deficit hyperactivity disorder (ADHD), language disorder, gastrointestinal symptoms, heart defects, and feeding problems [3]. Consequently, ASDs are increasingly understood to result from a variety of genetic variants that converge upon common biological pathways to impair brain development and produce a core set of diagnostic behavioral impairments. Stratification of ASD variants into subtypes defined by genetic etiology or affected biological pathway is the key to understanding the altered courses of neurodevelopment that occur in ASDs and for identifying targets of pharmacological therapy [4, 5].

Longitudinal study of early brain development in single gene disorders with high ASD penetrance is useful for linking known genetic and biological etiologies with abnormal neurodevelopment associated with ASD. Tuberous sclerosis complex (TSC) is one such disorder [4, 6]. TSC is caused by pathogenic variants in the *TSC1* or *TSC2* genes that encode suppressors of mechanistic target of rapamycin complex 1 (mTORC1). MTORC1 is a protein complex that regulates metabolic processes essential to cell growth. Failure of TSC1 and TSC2 to suppress mTORC1 results in mTORC1 hyperactivation, and subsequent growth of benign hamartomas in multiple organ systems, including the lungs, kidney, eyes, skin, heart, and brain [7]. In the brain, TSC neuropathology is characterized by altered cellular morphology, aberrant neuronal migration and proliferation, hypomyelination, gliosis, and disruption of laminar architecture [8]. These abnormalities are most concentrated in benign hamartomas, known as cortical tubers, that are distributed along the cortical-white matter interface and visible on MRI. Diffuse pathology, remote from tubers, exists as well [9, 10] and is detectable with diffusion tensor MRI [11–13]. Neurological impairments including ASD, epilepsy, intellectual disability, and ADHD are often associated with TSC, with variable severity [14].

TSC is amenable to prospective study of ASD neurodevelopment from birth because 40–50% of TSC patients develop an ASD [5, 15], TSC is typically diagnosed in utero or within the first year of life, prior to the emergence of ASD symptoms, and the genetic and biological underpinnings of TSC are well understood [16]. Further, TSC neuropathology is stochastically distributed throughout the brain, disrupting multiple brain circuits, and therefore provides an MRI-detectable pathological substrate for the “developmental disconnection” model of ASD. In this model, patients with ASD fail to develop appropriate connectivity between higher order cortical regions, resulting in global underconnectivity [17]. This model is appealing because it suggests a systems level, global deficit that could arise from aberration of a variety of neural mechanisms and genetic variants and thus is consistent with the genetic and phenotypic heterogeneity of ASDs [18]. MRI of children, adolescents, and adults with ASD has played a key role in the formulation of this model. Recurrent diffusion-weighted MRI findings of reduced microstructural integrity of long-range white matter fiber bundles as well as repeated reports of functional hypoconnectivity suggest underconnectivity from childhood onward in ASD [19, 20]. In TSC, children and adolescents with ASD exhibit reduced microstructural integrity of the corpus callosum, a mediator of interhemispheric connectivity across a wide array of functionally distinct brain regions [12, 21], and the arcuate fasciculus, a white matter pathway key to social communication [22], compared to those without ASD. Further study of other white matter structures in TSC and ASD is needed.

A caveat to the MRI ASD literature is that many of the studies report on children and adolescents and therefore describe the brain many years after the onset of the core behavioral features of ASD. These studies fail to describe the course of abnormal brain development that precedes and co-occurs with the onset of ASD symptoms. Prospective, longitudinal studies of neurodevelopment from birth through 3 years of age, or the period when ASD behaviors emerge, are needed to understand the series of early neurodevelopmental events that result in underconnectivity in childhood and beyond. Identification of brain regions that are first abnormal may improve our understanding of the biological mechanisms at play, provide pharmacological targets, improve diagnostic capability, and perhaps uncover a causal series of events that lead to underconnectivity and ASD behaviors.

Our purpose was to evaluate the relationship between white matter development over the first 2 years of life and ASD outcome at 24 months of age in TSC. We recruited TSC infants within their first year of life and imaged them with diffusion tensor imaging (DTI) at time of recruitment, 12 months of age, and at 24 months of age. At 24 months, subjects were diagnosed with or without ASD. We hypothesize that infants who go on to develop ASD exhibit global underconnectivity, or poor microstructural integrity of white matter, over the first 2 years of life compared to those who do not develop ASD.

## Methods

### Study design

This research was conducted under a prospective, ongoing, multisite TSC Autism Center for Excellence Research Network (TACERN) study investigating the developmental precursors of ASD in TSC via longitudinal clinical MRI, EEG, cognitive, and behavioral assessment. Infants were recruited and longitudinally evaluated at one of five TACERN sites, each with a TSC specialty clinic: Boston Children’s Hospital (BCH), Cincinnati Children’s Hospital Medical Center (CCHMC), University of Alabama at Birmingham (UAB), University of California Los Angeles (UCLA), and McGovern Medical School at University of Texas Health Science Center at Houston (UTH). All study procedures were approved by the Institutional Review Board at each site, and written informed consent was obtained.

Infants were enrolled between 3 and 12 months of age following diagnosis with TSC [16]. Diagnosis was based on genetic or clinical diagnostic criteria for TSC, including physical exam, neuroimaging, or echocardiogram. Exclusion criteria included history of gestational age < 36 weeks, exposure to mTOR inhibitor such as rapamycin (sirolimus) or everolimus, exposure to an investigational drug within 30 days of study enrollment, subependymal giant cell astrocytoma requiring medical or surgical treatment, neurosurgery, and contraindications for MRI.

**Fig. 1:**
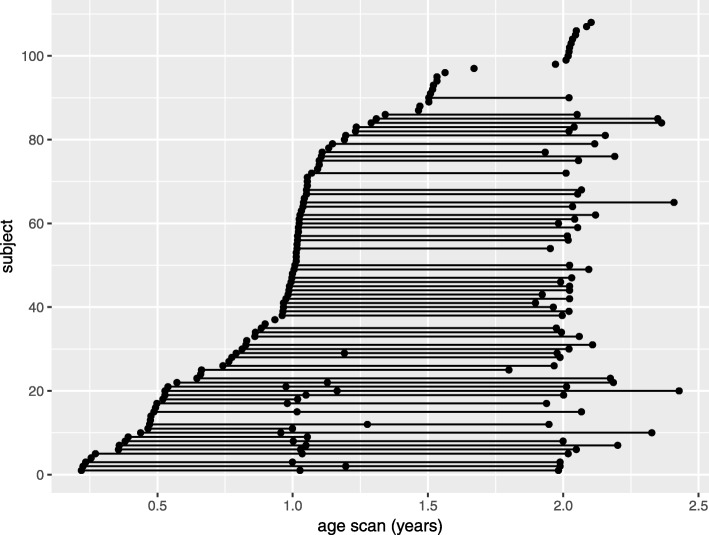
Depiction of the MRI sample. Each point represents an MRI scan. A line connecting multiple points represents repeated MRI scans for a single patient

Brain MRI was acquired at baseline, or time of enrollment, and at 12, 24, and 36 months of age, modified as required by clinical care demands. Because infants were enrolled between 3 and 12 months of age, age at baseline MRI varies across the cohort (Fig. 1). To allow a minimum 6–9 months between MRI scans, subjects with baseline MRI between 6 and 9 months of age were permitted to perform the 12-month MRI anytime between 12 and 15 months of age. If infants were enrolled between 10 and 12 months of age, the baseline MRI was foregone, and the first study MRI occurred at 12 months of age, followed by 24- and 36-month MRI scans. All MRI scans were sent to the Computational Radiology Laboratory at BCH for quality control and image processing.

Infants underwent developmental and clinical evaluations at 3, 6, 9, 12, 18, 24, and 36 months of age. The developmental evaluation consisted of standardized neuropsychological evaluation with adaptive and developmental measures by research-reliable personnel. The clinical evaluation entailed collection of baseline demographic information, baseline and interval medical history, family history, prior and concomitant medications, genetic data, clinical exam findings, and past and current seizure history. All developmental and clinical data were sent to the centralized TACERN Data Coordinating Center at UAB. A yearly calibration meeting was held to ensure developmental assessment reliability across all sites for the entire study period.

### MRI acquisition

Patient brain MRI scans were acquired at 3T on seven scanners and five scanner models, including one General Electric (GE) Signa HDxt, one Philips Achieva, three Philips Ingenia, one Siemens Skyra, and two Siemens TrioTim with 32, 12, and 8 channel head coils. Subjects were imaged under the TACERN consensus research imaging protocol that includes high resolution, routine clinical imaging sequences used for annual surveillance imaging of TSC patients. The protocol includes a 1-mm^3^ sagittal T1-weighted (T1w) MPRAGE, 0.4-mm^2^ in-plane resolution × 2-mm slice thickness axial T2-weighted (T2w) TSE, 30 high angular resolution *b* = 1000 s/mm^2^ diffusion-weighted (DW) images, and 6 *b* = 0 s/mm^2^ DW 2 mm^3^ resolution images, one with reversed phase-encoding direction for distortion compensation, covering the entire brain. Imaging protocols were harmonized to the extent permitted by each platform. Detailed acquisition parameters used on each scanner and cross-scanner reliability are detailed in a previous publication (Prohl 2019, under review) and in Additional file 1: Table S1. Patients were imaged under sedation or in natural sleep as clinically indicated.

### Quality assurance

MRI data were evaluated at the TACERN MRI Processing Center at the Computational Radiology Lab at BCH. MRI metadata were reviewed for protocol compliance. All image volumes were reviewed slice by slice by an expert rater for extent of brain coverage and artifacts resulting from a variety of sources, including but not limited to table vibration, magnetic susceptibility, subject motion, flow, radiofrequency leak, and venetian blind artifact [23, 24]. Diffusion-weighted volumes with artifact were removed prior to analysis.

### MRI processing

All MRI processing and analyses were completed using the Computational Radiology Kit (http://crl.med.harvard.edu) via a fully automated processing pipeline. In the native space of each scan, the T2w image was aligned and resampled to the 1 mm^3^ T1w image using rigid registration with mutual information metric. The intracranial cavity (ICC) was then segmented using a previously validated multispectral ICC segmentation method [25], and the ICC was masked from the T1w and T2w images.

The DW images were corrected for magnetic susceptibility distortion using the pair of *b* = 0 images with opposite phase-encoding direction and FSL topup [26]. Inter-volume motion correction was then performed by affine registration of each DW image to the average *b* = 0 s/mm^2^ image. The DW images were aligned and up-sampled to the 1 mm^3^ T1w scan using affine registration and sinc interpolation, and the brain extracted on DWI using the previously computed ICC segmentation [27]. A single tensor diffusion model was estimated using robust least squares in each brain voxel from which fractional anisotropy (FA = 3Var(*λ*)/(*λ*^2^_1_ + *λ*^2^_2_ + *λ*^2^_3_)^1/2^) and mean diffusivity (MD = (*λ*_1_ + *λ*_2_ + *λ*_3_)/3) were computed [28].

### Automatic ROI delineation

Next, a fully automatic, multi-template approach was used to define 17 white matter regions of interest (ROIs) in the native space of each subject DTI scan using a previously validated method [29]. A template library was constructed from whole brain DTI of 20 healthy children, with each scan in its native space. The DTI were computed from 30 high angular resolution *b* = 1000 s/mm^2^ and 5 *b* = 0 s/mm^2^ TACERN protocol DW images acquired on a 3T Siemens Skyra scanner at BCH.

For each template, scalar FA and color maps of the principal diffusion directions were computed from the DTI. ROIs were hand drawn by an expert rater on the color map within white matter fiber bundles following previously defined and validated labeling schemes for tractography [30–32]. To delineate the same white matter ROIs in the native space of each subject scan, the following procedure was performed for every template: the template scalar FA map was aligned to the target subject scalar FA map using affine registration with mutual information metric. The affine registration field was used to initialize a non-linear, dense registration of the template DTI to the subject DTI, for a total of 20 non-linear dense registrations per scan, and 3740 non-linear dense registrations in the sample of 187 MRI scans. The affine and dense deformation fields were then used to resample the template white matter ROIs to the subject native DTI space using nearest neighbor interpolation. Now with 20 sets of white matter ROIs (one for each template) aligned to the native space of the subject scan, a final, consensus set of white matter ROIs was computed using the STAPLE algorithm [33]. Lastly, mean FA and MD were computed in each ROI.

### White matter ROIs

**Fig. 2:**
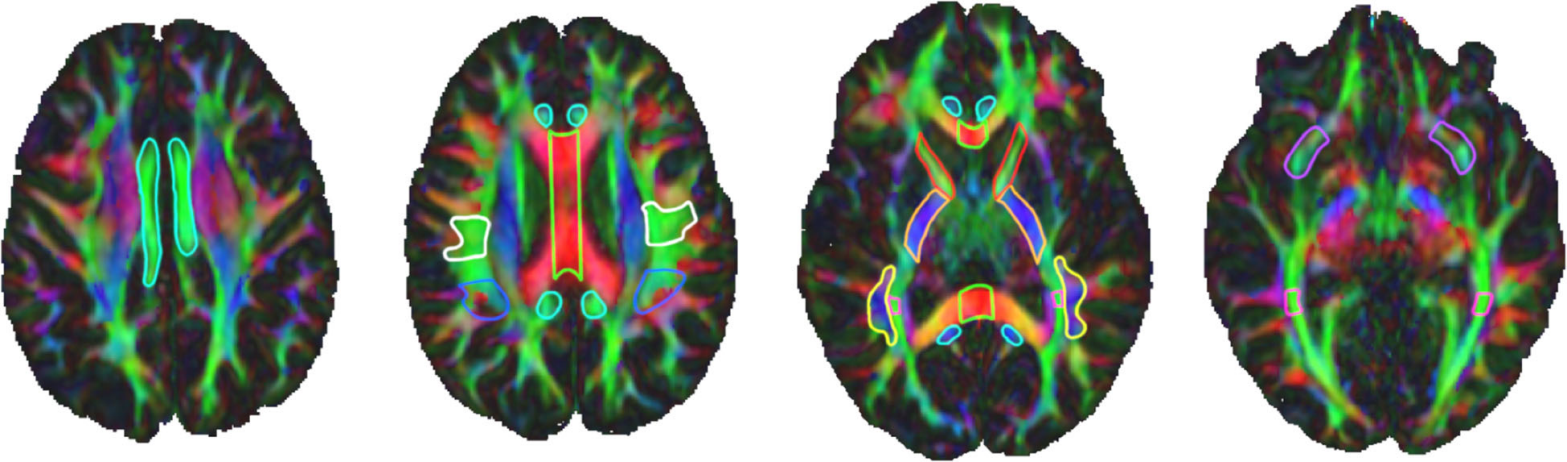
White matter regions of interest (ROI) superimposed on a color map of the principal diffusion directions. Red color map voxels indicate left-right diffusion, green color map voxels indicate anterior-posterior diffusion, blue color map voxels indicate inferior-superior diffusion, and other colors indicate intermediate diffusion directions. Four axial slices from a single scan depict 2D slices of 3D white matter ROI, outlined in unique colors: light blue = cingulum, green = corpus callosum, white = arcuate fasciculus waypoint to Broca’s; royal blue = arcuate fasciculus Geschwind’s territory, red = anterior limb of the internal capsule, orange = posterior limb of the internal capsule, yellow = arcuate fasciculus Wernicke’s area, pink = sagittal stratum, and purple = uncinate fasciculus

The ROIs analyzed in this analysis were defined using previously validated labeling schemes for tractography and include left and right posterior limb of the internal capsule, anterior limb of the internal capsule, cingulum body, inferior extreme capsule, and corpus callosum following [30]. Briefly, the ALIC and PLIC ROIs were drawn in the axial plane. The inferior limit of the ROIs was defined on the first axial slice superior to the anterior commissure, and the superior limit was defined on the axial slice where the lenticular nucleus separates the internal and external capsules. The cingulum was defined in the axial plane with a single ROI in each hemisphere covering the cingulum body. The inferior anterior limit of the ROI was defined in plane with the inferior genu of the corpus callosum, and the inferior poster limit was defined in plane with the inferior splenium of the corpus callosum. The superior ROI limit was defined on the most superior green fibers of the cingulum. The inferior extreme capsule ROIs were defined in the coronal plane. The posterior limit of the ROI was defined by the first slice anterior to the apex of UF curvature and covered ten slices of green-blue, fronto-temporal fibers anterior to the posterior limit. This approach deviates from the approach taken in Catani 2008, but produced accurate and reliable tractography and therefore was implemented. The corpus callosum ROI was drawn in the sagittal plane and covered the body of the corpus callosum with 10 mid sagittal slices (5 slices in each hemisphere) [30]. The sagittal stratum was defined in the coronal plane following the labeling technique for tractography of the optic radiation, presented in [32]. Briefly, the anterior limit of the ROI was defined on the coronal slice immediately posterior to the splenium of the corpus callous and the extended posteriorly for a total of 5 coronal slices. All green fibers were labeled with the purpose of capturing the optic radiations [32]. The arcuate fasciculus ROIs were placed following the labeling scheme presented in [22]. Three ROIs were placed along the arcuate fasciculi in each hemisphere; in the white matter (1) projecting from the inferior parietal lobule to the inferior frontal gyrus, (2) underlying the inferior parietal lobule, and (3) underlying the posterior superior temporal gyrus. From here on, we refer to these ROIs as left and right arcuate fasciculus waypoint to Broca’s, Geschwind’s Territory, and Wernicke’s area, respectively (Fig. 2).

### Developmental measures

The TACERN battery consists of multiple measures administered longitudinally that measure the extent of ASD symptomatology and developmental status of TSC infants [34]. Here, we focus on two of these measures administered at 24 months of age. First, the Autism Diagnostic Observation Schedule, Second Edition (ADOS-2) is a semi-structured, interactive observation schedule designed to assess individuals who may have an ASD and consists of 5 modules [35, 36]. The specific module (toddler, 1, or 2 were applicable to our cohort) was determined by the ADOS-2 administrator at the time of the assessment. Based on the overall total sum of selected items from the social affect and restricted and repetitive behavior domains, the Toddler Module yields classifications of little-to-no concern, mild-to-moderate concern, and moderate-to-severe concern for ASD and modules 1 and 2 yield classifications of non-spectrum, autism spectrum, or autism.

Second, the Mullen Scales of Early Learning (MSEL) was also completed at 24 months of age [37]. The MSEL provides an assessment of developmental functioning for children ages 0–5, with domain scores for fine and gross motor skills, visual reception, receptive and expressive language, and an overall early learning composite score. Developmental quotient (DQ) was used in lieu of the early learning composite score in order to capture the performance of low scoring subjects for whom standard scores were not available given their age and raw score. The DQ is equal to the average of fine motor, expressive language, receptive language, and visual reception developmental quotients. A DQ was computed for each domain and was equal to (domain age equivalent ÷ chronological age)× 100.

### Epilepsy measures

Parents recorded seizure types, seizure frequencies, and antiepileptic medications in a seizure diary over the study period, and data were collected at all clinical visits (3, 6, 9, 12, 18, 24, and 36 months of age). Epilepsy severity at 24 months of age was quantified by summing the number of seizure types and number of antiepileptic drugs used from 12 to 24 months of age. These two severity items were selected because they are good measures of epilepsy severity in TSC [38] and were available in all analyzed subjects.

### Statistical analysis

Statistics were completed using R version 3.5.1 and R Studio version 1.1.456 [39, 40]. Linear mixed effects (LME) modeling was performed to test the hypothesis that infants who develop ASD exhibit poor white matter microstructural integrity over the first 2 years of life compared to those who do not develop ASD. LME modeling is an appropriate method because it accommodates missing data and repeated measures. Missing data were not imputed and all available measurements were included under the missing-at-random assumption.

Longitudinal trajectories of FA and MD for each white matter ROI over the first 24 months of life were modeled using the lme4 package [41]. For each DTI metric (FA or MD) and in each white matter ROI, we computed a random intercept LME model in the natural logarithm of age, for a total of 34 models (17 white matter ROIs, 2 DTI metrics). Due to the rapid change in the brain microstructure in the first year of life, the natural logarithm of age at MRI scan was taken to linearize the model. Natural log of age at MRI scan was then broken into two components: (1) baseline age, or age at the subject’s first MRI scan in the sample, and (2) longitudinal age, or age at MRI scan minus baseline age. Age was split into these two components in order to separate the cross-sectional effect, which captures the relationship between DTI metrics and age at first MRI scan, from the longitudinal effect, which captures rate of change of DTI metrics and age [42]. In our sample, cross-sectional age (or age at first MRI scan) varied from 0.22 to 2.1 years of age, and thus, modeling the cross-sectional effect and longitudinal effect separately is appropriate [43].

In addition to baseline age and longitudinal age, other fixed effects of interest included group, sex, interaction of group with baseline age, and interaction of group with longitudinal age. To create a binary group variable (TSC-ASD, TSC+ASD) from the 24 month ADOS, infants were considered TSC-ASD if classified as little-to-no-concern on the Toddler Module or as non-spectrum on modules 1 and 2. Infants were considered TSC+ASD if classified as mild-to-moderate or moderate-to-severe-concern on the Toddler Module or as autism spectrum or autism on modules 1 and 2. Subject was modeled as a random intercept to capture between subject variability.

Likelihood ratio tests were used to evaluate the significance of each term to the model. Sex was dropped as it did not reach significance. For every ROI, the final model consisted of the following:

$$ {y}_{ij=}\ {\beta}_0+{\beta}_1{\mathrm{Group}}_i+{\beta}_2\mathrm{ag}{{\mathrm{e}}_{\mathrm{baseline}}}_i+{\beta}_3\mathrm{ag}{{\mathrm{e}}_{\mathrm{longitudinal}}}_{ij}+{\beta}_4\mathrm{Group}\times \mathrm{ag}{{\mathrm{e}}_{\mathrm{baseline}}}_i+{\beta}_5\mathrm{Group}\times \mathrm{ag}{{\mathrm{e}}_{\mathrm{longitudinal}}}_{ij}+{\vartheta}_{0i}+{\varepsilon}_{ij} $$

where *y* = FA or MD, *β* = fixed effect coefficient*, ϑ* = random effect coefficient*, i* indexes subject, and *j* indexes scan within each subject. I. The units of age_baseline and age_longitudinal are ln(years). The error term in the above model is assumed to identically independently distributed as *εij*~*N*(0, *σ*^2^), and the distribution of random-effects is assumed to be multivariate normal with mean 0 and variance covariance matrix Σ*ϑ*, i.e., (*ϑ*_0*i*_)~*N*(0, *Σ*_*ϑ*_).

### Sample

Inclusion criteria for the present analysis were (1) ADOS completed at 24 months of age and (2) one or more successful MRI scans with DWI. These criteria were met by 115 of 143 infants enrolled in the study. Two hundred eighty-eight scans were available from the 115 infants who met inclusion criteria. Of the 288 scans available for analysis, 76 scans collected at the 36-month time point were excluded, 18 scans were post-neurosurgical and therefore excluded, and 6 scans were excluded due to quality of the DWI. This yielded a sample of 108 subjects (62 TSC-ASD, 55% male; 46 TSC+ASD, 52% male) with 187 MRI scans (109 TSC-ASD; 78 TSC+ASD) for analysis (Table 1, Fig. 1). One hundred eighty-four of 187 scans featured no evidence of hardware or patient-induced artifact, and therefore, 30 high angular resolution *b* = 1000 s/mm^2^ DW images were processed. Two of 187 scans required removal of a single gradient volume due to patient motion. One of 187 scans required removal of 10 gradient volumes due to patient motion. One hundred one (94%) of subjects were scanned in a single scanner for all study visits. Seven subjects were scanned on 2 scanners over the course of their study visits. Sedation was used as clinically indicated in 186 of 187 scans.

**Table 1:** Descriptive data of the sample by diagnostic outcome group

	TSC-ASD	TSC+ASD	*t*	*p*
Subjects (*n*, %)	62, 57%	46, 43%		
Sex (% male)	55%	52%		
MSEL developmental quotient at 24 months ($\overline{x}$ ± *σ*)	90.4 ± 19.8	61.5 ± 19.7	7.6	1.7 × 10− ^11^
Epilepsy Severity Score at 24 months ($\overline{x}$ ± *σ*)	2.1 ± 1.6	3.6 ± 1.8	− 4.8	6.1 × 10− ^6^
ADOS-2 calibrated severity score ($\overline{x}$ ± *σ*)	1.7 ± 0.8	6.4 ± 2.0	− 15.5	< 2.2 − 10^−16^

*MSEL* Mullen Scales of early learning, *ADOS* autism diagnostic observation schedule

## Results

### Descriptive statistics

Descriptive data of the sample by diagnostic outcome group is available in Table 1. Subjects with ASD exhibited a significantly lower MSEL DQ (*t* = 7.6, *p* = 1.7 × 10^−11^) and significantly higher epilepsy severity score (*t* = ^−^4.8, *p* = 6.1 × 10^−6^) at 24 months of age compared to TSC subjects without ASD (Table 1). Subjects with ASD had greater severity of ASD symptoms overall as measured by the ADOS calibrated severity score, as expected (*t* = ^−^15.5, *p* < 2.2 × 10^−16^). Descriptive data of the MRI sample by diagnostic outcome group is available in Table 2. The distribution of scans across baseline, 12 months, and 24 months of age was similar between groups. In subjects with ASD, 15% of scans were acquired at baseline, 43% of scans were acquired at 12 months, and 42% of scans were acquired at 24 months. In subjects without ASD, 19% of scans were acquired at baseline, 40% of scans were acquired at 12 months, and 41% of scans were acquired at 24 months. There were no group differences in age at 12-month or 24-month MRI scan. Subjects with ASD were imaged at baseline significantly later (0.6 ± 0.2 years) than subjects without ASD (0.4 ± 0.2) (*t* = ^−^2.1, *p* = 0.04).

**Table 2:** Descriptive data of the MRI sample by diagnostic outcome group

	TSC-ASD	TSC+ASD
Scans (subjects) with ADOS	163 (66)	125 (49)
Excluded: 36-month scans (*n*)	44	32
Excluded: Post-surgical scans (*n*)	5	13
Excluded: DWI artifact scans (*n*)	4	2
Total scans (subjects) in sample	109 (62)	78 (46)
Sedated scans (*n*, %)	109, 100%	77, 99%
Baseline MRI (*n*, %)	16, 15%	15, 19%
12 months MRI (*n*, %)	47, 43%	31, 40%
24 months MRI (*n*, %)	46, 42%	32, 41%
Age at baseline MRI ($\overline{x}$ ± *σ*)	0.4 ± 0.2	0.6 ± 0.2
Age at 12 months MRI ($\overline{x}$ ± *σ*)	1.1 ± 0.1	1.1 ± 0.2
Age at 24 months MRI ($\overline{x}$ ± *σ*)	2.0 ± 0.2	2.0 ± 0.2
Subjects with 1 scan (*n*, %)	25, 40%	20, 44%
Subjects with 2 scans (*n*, %)	26, 42%	20, 43%
Subjects with 3 scans (*n*, %)	11, 18%	6, 13%

### Longitudinal mixed effects models: trajectories of white matter development

To test the hypothesis that white matter maturation varied as a function of diagnostic outcome group (TSC+ASD or TSC-ASD), a random intercept mixed effects model was computed for each DTI metric (FA and MD) in each white matter ROI, for a total for 34 models. For all white matter regions, FA significantly increased with baseline age and longitudinal age, and MD significantly decreased with baseline age and longitudinal age, as expected (Additional file 1: Table S2 and Table S3).

**Fig. 3:**
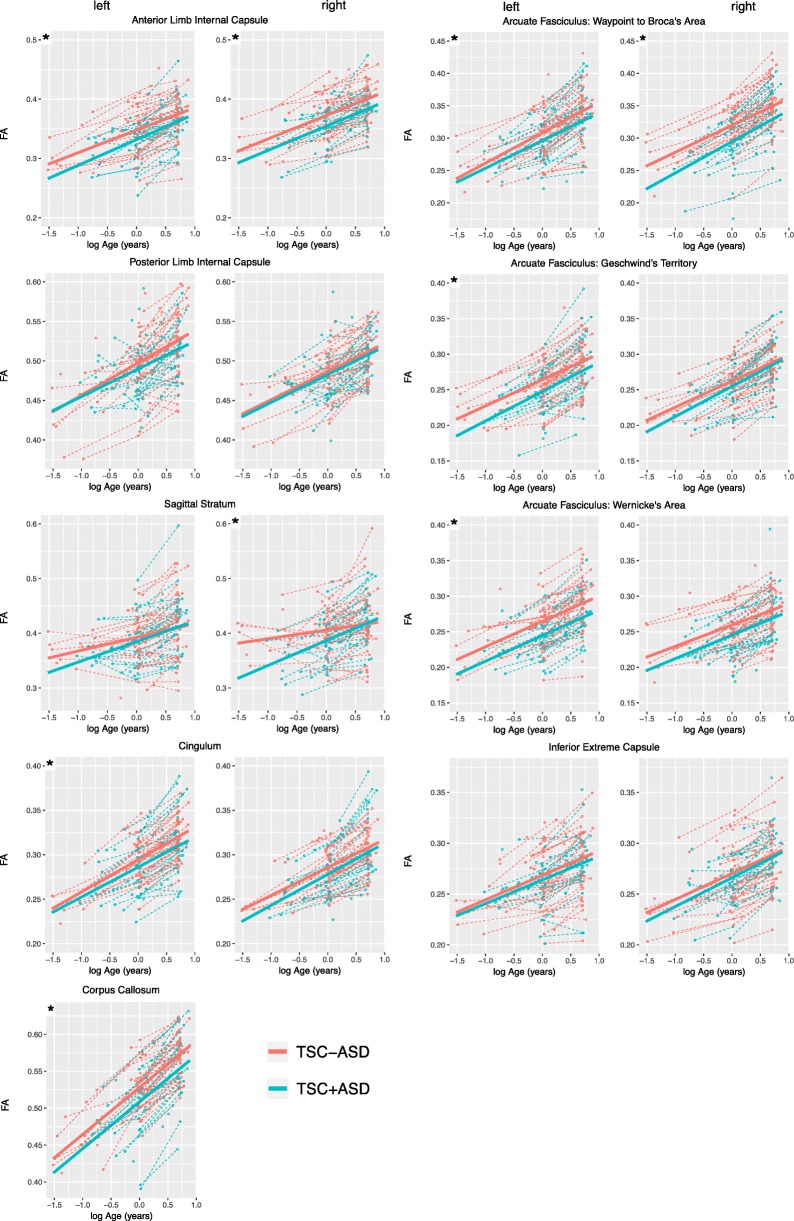
Fractional anisotropy of white matter regions of interest plotted as a function of natural logarithm of age in the TSC+ASD and TSC-ASD groups. Points represent MRI scans. Dashed lines represent raw FA trajectories for each subject. Solid lines represent the mean LME model fit

In all white matter regions, the main effect of group reduced FA, indicating that FA trajectories were lower in TSC+ASD compared to TSC-ASD. This effect of group on FA reached statistical significance in 9 of 17 white matter regions analyzed: left arcuate fasciculus waypoint to Broca’s (*χ*^2^ (1) = 6.07, *p* = 0.01), left arcuate fasciculus Geschwind’s territory (*χ*^2^ (1) = 5.62, *p* = 0.02), left arcuate fasciculus Wernicke’s area (*χ*^2^ (1) = 12.41, *p* < 0.001)), right arcuate fasciculus waypoint to Broca’s area (*χ*^2^ (1) = 11.42, (*p* < 0.001), left anterior limb internal capsule (*χ*^2^ (1) = 4.42, *p* = 0.04), right anterior limb internal capsule (*χ*^2^ (1) = 9.22, *p* = 0.002), left cingulum (*χ*^2^ (1) = 4.48, *p* = 0.03), corpus callosum (*χ*^2^ (1) = 11.66, *p* < 0.001), and right sagittal stratum (*χ*^2^ (1) = 6.55, *p* = 0.01) (Table 3, Fig. 3).

**Table 3:** Longitudinal mixed effects model results for effect of group (TSC+ASD and TSC-ASD) and group interactions with age on fractional anisotropy of white matter regions. Likelihood ratio tests were used to attain *p* values. Italicized *p* values indicate *p* < 0.05

	White matter region of interest	Group			Group × baseline age			Group × longitudinal age		
		Est	*χ*^2^	*p*	Est	*χ*^2^	*p*	Est	*χ*^2^	*p*
Left	Arcuate: waypoint to Broca’s	− 0.018	6.07	*0.01*	− 0.017	1.40	0.24	0.006	1.01	0.32
	Arcuate: Geschwind’s territory	− 0.016	5.62	*0.02*	0.004	0.08	0.78	0.004	0.96	0.33
	Arcuate: Wernicke’s area	− 0.023	12.41	*<0.001*	− 0.006	0.24	0.62	0.005	1.10	0.30
	Anterior limb internal capsule	− 0.015	4.42	*0.04*	0.01	0.47	0.49	0.002	0.21	0.65
	Cingulum	− 0.011	4.48	*0.03*	− 0.009	0.66	0.42	0.002	0.27	0.61
	Inferior extreme capsule	− 0.006	1.18	0.28	− 0.002	0.02	0.88	0.001	0.03	0.86
	Posterior limb internal capsule	− 0.009	1.46	0.23	− 0.005	0.14	0.71	− 0.01	0.53	0.47
	Sagittal stratum	− 0.003	0.10	0.75	0.023	1.45	0.23	0.004	0.22	0.64
Midline	Corpus callosum	− 0.024	11.66	*<0.001*	− 0.008	0.39	0.54	0.007	1.63	0.20
Right	Arcuate: waypoint to Broca’s	− 0.024	11.42	*<0.001*	0.006	0.19	0.66	0.006	1.56	0.21
	Arcuate: Geschwind’s territory	− 0.004	0.42	0.52	0.013	1.12	0.29	− 0.000	0.02	0.90
	Arcuate: Wernicke’s area	− 0.013	3.97	0.05	0.004	0.08	0.78	0.002	0.26	0.61
	Anterior limb internal capsule	− 0.020	9.22	*0.002*	0.001	0.01	0.94	0.003	0.34	0.56
	Cingulum	− 0.010	3.94	0.05	0.000	0.00	0.99	0.007	3.05	0.08
	Inferior extreme capsule	− 0.002	0.16	0.69	0.010	0.92	0.34	− 0.002	0.49	0.48
	Posterior limb internal capsule	− 0.003	0.31	0.58	0.003	0.06	0.81	− 0.002	0.13	0.72
	Sagittal stratum	− 0.022	6.55	*0.01*	0.023	1.78	0.18	0.035	13.48	*<0.001*

Italicized *p* values indicate *p* < 0.05

The interaction of baseline age and group did not significantly affect FA in any white matter regions. The interaction of group and longitudinal age significantly decreased FA in the right sagittal stratum only, indicating that with age, FA of the right sagittal stratum diverges between TSC+ASD and TSC-ASD, with TSC+ASD exhibiting a reduced FA over time (*χ*^2^ (1) = 13.48, *p* < 0.001) (Table 3).

**Fig. 4:**
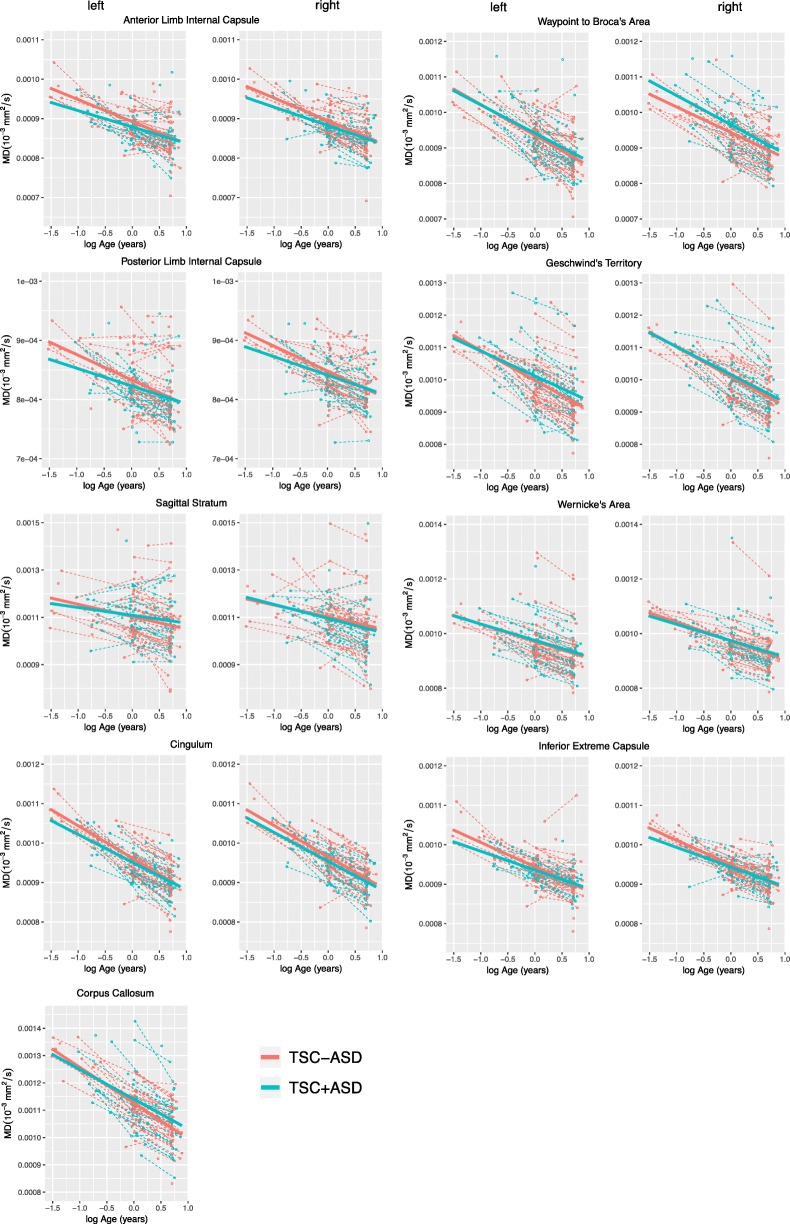
Mean diffusivity of white matter regions of interest plotted as a function of natural logarithm of age in the TSC+ASD and TSC-ASD groups. Points represent MRI scans. Dashed lines represent raw FA trajectories for each subject. Solid lines represent mean LME model fit.

In contrast to FA, the main effect of group did not significantly affect MD in any white matter regions. In all bilateral arcuate ROI, bilateral sagittal stratum, and the corpus callosum, the main effect of group increased MD, indicating that MD trajectories were higher in TSC+ASD compared to TSC-ASD; however, none of these effects were statistically significant. In the bilateral anterior limb internal capsule, bilateral posterior limb internal capsule, bilateral cingulum, and bilateral inferior extreme capsule, MD trajectories were higher in TSC-ASD compared to TSC+ASD, although none of these effects were significant (Table 4, Fig. 4).

**Table 4:** Longitudinal mixed effects model results for effect of group (TSC+ASD and TSC-ASD) and group interactions with age on mean diffusivity of white matter regions. Likelihood ratio tests were used to attain *p* values. Italicized *p* values indicate *p* < 0.05. Model estimates are scaled × 1000

	White matter region of interest	Group			Group × baseline age			Group × longitudinal age		
		Estimate	*χ*^2^	*p*	Estimate	*χ*^2^	*p*	Estimate	*χ*^2^	*p*
Left	Arcuate: waypoint to Broca’s	0.011	0.71	0.40	0.023	0.78	0.38	− 0.004	0.11	0.74
	Arcuate: Geschwind’s territory	0.029	3.01	0.08	0.052	2.40	0.12	− 0.013	1.43	0.23
	Arcuate: Wernicke’s area	0.005	0.11	0.74	0.015	0.22	0.64	− 0.004	0.17	0.68
	Anterior limb internal capsule	− 0.009	0.70	0.40	0.024	1.14	0.29	0.007	0.71	0.40
	Cingulum	− 0.005	0.46	0.50	0.025	2.41	0.12	0.002	0.04	0.84
	Inferior extreme capsule	− 0.005	0.39	0.53	0.020	1.67	0.20	0.010	1.34	0.25
	Posterior limb internal capsule	− 0.006	0.32	0.57	0.025	1.43	0.23	0.004	0.18	0.67
	Sagittal stratum	0.004	0.03	0.85	0.019	0.19	0.67	0.021	1.64	0.20
Midline	Corpus callosum	0.028	2.97	0.08	0.049	2.25	0.13	− 0.002	0.02	0.88
Right	Arcuate: waypoint to Broca’s	0.025	3.82	0.05	0.002	0.01	0.93	− 0.018	2.58	0.11
	Arcuate: Geschwind’s territory	0.008	0.24	0.63	0.012	0.15	0.70	0.002	0.04	0.84
	Arcuate: Wernicke’s area	0.006	0.18	0.68	0.023	0.61	0.43	− 0.003	0.08	0.78
	Anterior limb internal capsule	− 0.009	1.07	0.30	0.017	0.93	0.34	0.006	0.47	0.49
	Cingulum	− 0.007	0.81	0.37	0.018	1.30	0.25	− 0.004	0.22	0.64
	Inferior extreme capsule	− 0.006	0.72	0.39	0.011	0.68	0.41	0.012	2.22	0.14
	Posterior limb internal capsule	− 0.004	0.23	0.63	0.017	0.98	0.32	0.006	0.74	0.39
	Sagittal stratum	0.022	0.88	0.35	0.045	0.97	0.32	− 0.056	4.17	*0.04*

Italicized *p* values indicate *p* < 0.05

The interaction of baseline age and group did not significantly affect MD in any white matter regions. The interaction of group and longitudinal age significantly decreased MD in the right sagittal stratum only, indicating that with age, MD of the right sagittal stratum diverges between TSC+ASD and TSC-ASD, with TSC+ASD exhibiting a reduced MD over time (*χ*^2^ (1) = 4.17, *p* = 0.04) (Table 4).

## Discussion

We carried out a large prospective longitudinal study of TSC subjects. We assessed white matter maturation over the first 24 months of life and compared the trajectories of white matter maturation in subjects with and without ASD. In 9 of 17 white matter regions evaluated, TSC+ASD subjects exhibited disrupted microstructural integrity of the white matter compared to TSC-ASD subjects. The regions were the arcuate fasciculi, corpus callosum, cingulum, sagittal stratum, and anterior limb of the internal capsule. These results suggest that underconnectivity across multiple white matter fiber bundles develops prior to and concurrent with the emergence of ASD behavioral features over the first 2 years of life.

We also found subjects who were later diagnosed with ASD had higher severity of epilepsy and increased intellectual disability. Therefore, some of the changes in white matter properties may be associated with the higher incidence of epilepsy or the higher incidence of intellectual disability. Indeed, previous work has found the effects of ASD, epilepsy, and IQ on white matter microstructural abnormality to be additive; the more neurological comorbidities, the more abnormal the white matter, with ASD diagnosis contributing the most to abnormal white matter microstructure [21]. Given the widespread distribution of disease burden in TSC that affects multiple white matter pathways and therefore multiple brain functions, it follows that patients with greater overall neuropathological burden are at greater risk for impaired social functioning as well as impaired functioning in other domains. Larger studies with more subjects, exhibiting a full range of epilepsy severity and developmental functioning, are needed to assess the contribution of white matter maturation to each outcome.

### Fronto-temporal and fronto-limbic pathways

Four of the six fronto-temporal/fronto-limbic fiber bundles examined exhibited reduced FA in ASD. FA was reduced in all left arcuate regions and in the waypoint to Broca’s region on the right, compared to subjects without ASD. These findings indicate that previously reported abnormality of the arcuate in children and adults with TSC+ASD [22] originates early in the brain development. Reduced arcuate FA [19] and reduced FA of white matter underlying the temporoparietal junction [44–46] is also reported in children and adults with nsASD compared to controls. These findings are noteworthy because the AF supports temporoparietal cortex involved in social communication. The bilateral posterior STG mediate audition and phoneme discrimination within the first 6 months of life and play a key role in detecting speech prosody [47]. A 12-month-old infants later diagnosed with ASD exhibit reduced sensitivity to human voices and deficits in expressive and receptive language [15, 48, 49] compared to low-risk controls. The posterior superior temporal sulci are also implicated in the analysis of dynamic, biologically relevant stimuli, including eye-gaze, facial expressions, and body movements [50, 51]. During gaze and facial processing tasks and tasks of joint attention [52], the posterior STS is consistently reported to function atypically in adults and children with ASD compared to typically developing controls. Critically, acquisition of joint attention, a behavior dependent on biological motion perception, is thought to set the stage for development of complex social communication behaviors, such as language [53]. Delays in joint attention within the first 18 months of life predict later language deficits at 24 months of age in ASD [54, 55]. Our results contribute to mounting evidence implicating abnormality of the temporoparietal junction in ASD.

The uncinate fasciculus has also been implicated in joint attention and communication. The UF connects the orbitofrontal cortex with the anterior temporal lobe and has been proposed to play a role in joint attention [56], social-emotional processing [57], semantic retrieval, and sound recognition [58]. Infants with non-syndromic ASD exhibit reduced UF FA from 6 to 24 months and in child and adulthood [59, 60]. Here, we did not find atypical maturation in the inferior extreme capsule ROIs, which lie along the uncinate fasciculus, associated with ASD. This may reflect the positioning of the ROI at the outflow of the temporal stem to the frontal lobe, which includes both UF streamlines and streamlines of the inferior occipitofrontal fasciculus that lies just superior to the UF [61]. Future tractography analysis of the full UF tract will better define the relationship between the UF and TSC+ASD.

FA was also reduced in the left cingulum of subjects with ASD and approached statistically significant reduction in the right cingulum (*p* = 0.05). Reduced cingulum FA has been reported in children and adults with nsASD [60, 62] and has not been previously studied in infants with ASD. The cingulum connects medial aspects of the cingulate cortex with medial frontal, parietal, and temporal lobes and is predominately comprised of short U fibers that interlink these regions. The cingulum is responsible for a variety of functions related to emotion, motivation, executive function, and memory [62]. The cingulum is also thought to mediate and connection between the anterior and posterior hubs of the default mode network, a functionally defined network frequently identified as abnormal in ASD. From 6 to 24 months of age, atypical default mode network connectivity is associated with ASD [63] and restricted to repetitive behaviors [64]. Our findings in the cingulum provide a plausible neural substrate for atypically connectivity within the default mode network in ASD.

### Projection pathways

Three of the six projection fiber bundles examined exhibited reduced FA in ASD. FA was reduced in the ALIC bilaterally, a finding also reported in infants and children with nsASD [59, 65, 66]. The ALIC contains projection fibers from the thalamus, which comprise the anterior thalamic radiation, and projection fibers from the brainstem. The thalamus is a hub of sensorimotor connectivity, and atypical thalamocortical connectivity has oft been associated with ASD [67]. Thus, our findings of bilateral reduced ALIC FA are in line with previous reports of ALIC underconnectivity. We did not find group differences in the posterior limb of the internal capsule.

### Commissural pathways

We found reduced FA of the corpus callosum associated with TSC+ASD. This finding aligns with previous reports of reduced CC FA in children and adults with TSC+ASD [12, 21], children and adults with nsASD [19], and infants with nsASD [59]. The primary role of the CC is to mediate interhemispheric connectivity, and it is attributed functions of complex processing, working memory, and overall cognition. Corpus callosum abnormalities are the most consistently reported finding in nsASD DTI literature; however, a consistent link between atypical social functioning and corpus callosum microstructure has not been established [19]. This may be due to the diversity of functions of the corpus callosum, as well as the heterogeneity of the autism symptom profile. One recent study in nsASD over the first 24 months of life found an association between microstructure of the corpus callosum genu and sensory responsiveness and restricted and repetitive behaviors, but not social functioning [68]. More studies that focus on brain-behavior relationships, rather than brain-diagnostic outcome relationships, are needed to identify how variation in brain structures is associated with variation in ASD symptoms.

There are methodological points to consider about this study. First, although a wealth of functional, EEG, and DTI data suggest disrupted connectivity in ASD [20, 69], the validity of these findings is sometimes questioned. Head motion artifact in particular has been proposed as the cause of group difference in ASD versus typically developing control DTI studies [70–73], as FA is known to substantial decrease with DWI slice-level motion artifact [74]. Here, we present longitudinal imaging data acquired in infants at high risk of ASD with a common genetic diagnosis over the period of early brain development. The data set features virtually no head motion artifact and stringent quality control for hardware-induced artifact. Thus, we consider this an ideal data set for linking atypical development of brain structures with the emergence of ASD-related behaviors.

Second, TSC+ASD was associated with reduced FA, but not elevated MD. FA reflects the degree of water anisotropy in the voxel, which is most strongly driven by the presence of axons. In contrast, MD is a measure of overall diffusivity and is highly sensitive to myelination [75]. Over the first 2 years of life, rapid myelination is occurring throughout the brain at different rates in different locations in different subjects. We hypothesize that the inter-subject variability in overall water diffusion within this age range is high due to these varying rates of maturation of myelin, or other white matter microstructure, and masks our ability to detect group differences in MD. FA group differences are detectable because the primary driver of FA is the presence of the axons, myelinated or not.

Third, longitudinal imaging was not available for all subjects analyzed. Ten subjects who met inclusion criteria underwent neurosurgery for epilepsy treatment, and longitudinal, presurgical imaging was not possible. Fourth, single tensor diffusion models are limited in regions of crossing fascicles. Multi-tensor model estimation ameliorates this limitation and also provides more specific pathological correlates of diffusion properties than single tensor models [76]. However, unlike multi-tensor models or any tractography-based analysis, our method is valuable for its clinical applicability. Region of interest analysis can be performed on scanner outputted FA maps in the absence of patient motion and are not dependent upon user-defined tracking parameters.

## Conclusion

We found evidence of white matter underconnectivity in multiple distinct brain regions over the first 2 years of life in TSC, a single gene disorder with high penetrance of ASD. Future studies examining brain-behavior relationships in TSC+ASD are warranted to improve our understanding of the neural substrates of ASD.

## Supplementary information

**Additional file 1: Table S1.** Clinical T1, T2, and Diffusion-weighted MR protocols 1 for the TACERN study. **Table S2.** Full longitudinal mixed effect model results for longitudinal trajectories of fractional anisotropy (FA) in each white matter ROI. **Table S3.** Full longitudinal mixed effect model results for longitudinal trajectories of mean diffusivity (MD) in each white matter ROI. Model estimates are scaled X 1000.

## Data Availability

The datasets analyzed during this study are available from the corresponding author on reasonable request.

## Abbreviations

ADHD: Attention-deficit hyperactivity disorder
ADOS: Autism Diagnostic Observation Schedule
ASD: Autism spectrum disorders
BCH: Boston Children’s Hospital
CCHMC: Cincinnati Children’s Hospital Medical Center
DQ: Developmental quotient
DTI: Diffusion tensor imaging
DWI: Diffusion-weighted imaging
FA: Fractional anisotropy
FOV: Field of view
GE: General electric
ICC: Intracranial cavity
LME: Linear-mixed effects
MD: Mean diffusivity
MRI: Magnetic resonance imaging
MSEL: Mullen Scales of Early Learning
MTORC1: Mechanistic target of rapamycin complex 1
PSTAPLE: Probabilistic simultaneous truth and performance level estimation
ROI: Region of interest
T1W: T1-weighted
T2W: T2-weighted
TACERN: Tuberous Sclerosis Complex Autism Center of Excellence Research Network
TE: Echo time
TR: Repetition time
TSC: Tuberous Sclerosis Complex
UAB: University of Alabama
UCLA: University of California Los Angeles
UTH: University of Texas Houston

## References

[1] Jones EJH, Gliga T, Bedford R, Charman T, Johnson MH (2014). Developmental pathways to autism: a review of prospective studies of infants at risk. Neurosci Biobehav Rev..

[2] Baio J, Wiggins L, Christensen DL, Maenner MJ, Daniels J, Warren Z (2018). Prevalence of autism spectrum disorders in a total population sample-autism and developmental disabilities monitoring network, 11 sites, United States, 2014. MMWR Surveill Summ..

[3] Vorstman JAS, Parr JR, Moreno-De-Luca D, Anney RJL, Nurnberger JI, Hallmayer JF (2017). Autism genetics: opportunities and challenges for clinical translation. Nat Rev Genet..

[4] Jeste SS, Geschwind DH (2014). Disentangling the heterogeneity of autism spectrum disorder through genetic findings. Nat Rev Neurol..

[5] De La Torre-Ubieta L, Won H, Stein JL, Geschwind DH (2016). Advancing the understanding of autism disease mechanisms through genetics. Nat Med..

[6] Davis PE, Peters JM, Krueger DA, Sahin M (2015). Tuberous sclerosis: a new frontier in targeted treatment of autism. Neurotherapeutics..

[7] Crino PB (2016). The mTOR signalling cascade: paving new roads to cure neurological disease. Nat Rev Neurol..

[8] Crino PB (2013). Evolving neurobiology of tuberous sclerosis complex. Acta Neuropathol..

[9] Ruppe V, Dilsiz P, Reiss CS, Carlson C, Devinsky O, Zagzag D (2014). Developmental brain abnormalities in tuberous sclerosis complex: a comparative tissue analysis of cortical tubers and perituberal cortex. Epilepsia..

[10] Marcotte L, Aronica E, Baybis M, Crino PB (2012). Cytoarchitectural alterations are widespread in cerebral cortex in tuberous sclerosis complex. Acta Neuropathol..

[11] Krishnan ML, Commowick O, Jeste SS, Weisenfeld N, Hans A, Gregas MC (2010). Diffusion features of white matter in tuberous sclerosis with tractography. Pediatr Neurol..

[12] Peters JM, Sahin M, Vogel-Farley VK, Jeste SS, Nelson CA, Gregas MC (2012). Loss of white matter microstructural integrity is associated with adverse neurological outcome in tuberous sclerosis complex. Acad Radiol..

[13] Peters JM, Prohl AK, Tomas-Fernandez XK, Scherrer B, Lidov HG, Singh JM (2015). Tubers are neither static nor discrete: evidence from serial diffusion tensor imaging. Neurology..

[14] Henske EP, Jóźwiak S, Kingswood JC, Sampson JR, Thiele EA (2016). Tuberous sclerosis complex. Nat Rev Dis Prim..

[15] Jeste S, Wu J, Senturk D, Varcin K, JKo J, McCarthy B (2014). Early developmental trajectories associated with ASD in infants with tuberous sclerosis complex. Neurology..

[16] Davis PE, Filip-Dhima R, Sideridis G, Peters JM, Au KS, Northrup H (2017). Presentation and diagnosis of tuberous sclerosis complex in infants. Pediatrics.

[17] Geschwind DH, Levitt P (2007). Autism spectrum disorders: developmental disconnection syndromes. Curr Opin Neurobiol..

[18] Pelphrey KA, Shultz S, Hudac CM, Vander Wyk BC (2011). Research review: constraining heterogeneity: the social brain and its development in autism spectrum disorder. J Child Psychol Psychiatry Allied Discip..

[19] Travers BGB, Adluru N, Ennis C, Tromp DPM, Destiche D, Doran S (2012). Diffusion tensor imaging in autism spectrum disorder: a review. Autism Res..

[20] Rane P, Cochran D, Hodge SM, Haselgrove C, Kennedy D, Frazier JA (2015). Connectivity in autism: a review of MRI connectivity studies. Harv Rev Psychiatry..

[21] Baumer FM, Peters JM, Clancy S, Prohl AK, Prabhu SP, Scherrer B, et al. Corpus callosum white matter diffusivity reflects cumulative neurological comorbidity in tuberous sclerosis complex. Cereb Cortex. 2017;(Md):1–8. 10.1093/cercor/bhx247 PMC6132277 29939236

[22] Lewis WW, Sahin M, Scherrer B, Peters JM, Suarez RO, Vogel-Farley VK (2013). Impaired language pathways in tuberous sclerosis complex patients with autism spectrum disorders. Cereb Cortex..

[23] Gallichan D, Scholz J, Bartsch A, Behrens TE, Robson MD, Miller KL (2010). Addressing a systematic vibration artifact in diffusion-weighted MRI. Hum Brain Mapp..

[24] Morelli JN, Runge VM, Ai F, Attenberger U, Vu L, Schmeets SH (2011). An image-based approach to understanding the physics of mr artifacts. RadioGraphics..

[25] Grau V, AUJ M, Alcañiz M, Kikinis R, Warfield SK (2004). Improved watershed transform for medical image segmentation using prior information. IEEE Trans Med Imaging..

[26] Andersson JLR, Skare S, Ashburner J (2003). How to correct susceptibility distortions in spin-echo echo-planar images: application to diffusion tensor imaging. Neuroimage..

[27] Dyrby TB, Lundell H, Burke MW, Reislev NL, Paulson OB, Ptito M (2014). Interpolation of diffusion weighted imaging datasets. Neuroimage..

[28] Mori S, Zhang J (2006). Principles of diffusion tensor imaging and its appolications in basic neuroscience research. Neuron..

[29] Suarez RO, Commowick O, Prabhu SP, Warfield SK (2012). Automated delineation of white matter fiber tracts with a multiple region-of-interest approach. Neuroimage..

[30] Catani M, Thiebaut de Schotten M (2008). A diffusion tensor imaging tractography atlas for virtual in vivo dissections. Cortex..

[31] Catani M, Jones DK, Ffytche DH (2005). Perisylvian language networks of the human brain. Ann Neurol..

[32] Benjamin CFA, Singh JM, Prabhu SP, Warfield SK (2014). Optimization of tractography of the optic radiations. Hum Brain Mapp..

[33] Warfield SK, Zou KH, Wells WM (2004). Simultaneous truth and performance level estimation (STAPLE). IEEE Trans Med Imaging..

[34] Capal JK, Horn PS, Murray DS, Byars AW, Bing NM, Kent B (2017). Utility of the autism observation scale for infants in early identification of autism in tuberous sclerosis complex. Pediatr Neurol..

[35] Lord C, Risi S, Linda L, Cook EH, Leventhal BL, Di Lavore PC (2000). The autism diagnostic observation schedule - generic: a standard mesure of social and communication deficits associated with the spectrum of autism. J Autism Dev Disord..

[36] Luyster R, Gotham K, Guthrie W, Coffing M, Petrak R, Pierce K (2009). The autism diagnostic observation schedule - toddler module: a new module of a standardized diagnostic measure for autism spectrum disorders. J Autism Dev Disord..

[37] Mullen E (1995). Mullen Scales of early learning (AGS ed.). AGS.

[38] Humphrey A, Ploubidis GB, Yates JRW, Steinberg T, Bolton PF (2008). The Early Childhood Epilepsy Severity Scale (E-Chess). Epilepsy Res..

[39] Team RC (2018). R: a language and environment for statistical computing.

[40] Rs T (2016). RStudio: Integrated Development for R.

[41] Bates D, Maechler M, Bolker B, Walker S (2015). Fitting linear mixed-effects models using lme4. J Stat Softw..

[42] Neuhaus J, Kalbfleisch J (1998). Between- and within-cluster covariate effects in the analysis of clustered data. Biometrics..

[43] Morrell CH, Brant LJ, Ferrucci L (2009). Model choice can obscure results in longitudinal studies. J Gerontol A Biol Sci Med Sci..

[44] Barnea-Goraly N, Kwon H, Menon V, Eliez S, Lotspeich L, Reiss AL (2004). White matter structure in autism: preliminary evidence from diffusion tensor imaging. Biol Psychiatry..

[45] Lee JE, Bigler ED, Alexander AL, Lazar M, DuBray MB, Chung MK (2007). Diffusion tensor imaging of white matter in the superior temporal gyrus and temporal stem in autism. Neurosci Lett..

[46] Noriuchi M, Kikuchi Y, Yoshiura T, Kira R, Shigeto H, Hara T (2010). Altered white matter fractional anisotropy and social impairment in children with autism spectrum disorder. Brain Res..

[47] Skeide MA, Friederici AD (2016). The ontogeny of the cortical language network. Nat Rev Neurosci..

[48] Estes A, Zwaigenbaum L, Gu H, St. John T, Paterson S, Elison JT (2015). Behavioral, cognitive, and adaptive development in infants with autism spectrum disorder in the first 2 years of life. J Neurodev Disord.

[49] Sperdin HF, Schaer M (2016). Aberrant development of speech processing in young children with autism: new insights from neuroimaging biomarkers. Front Neurosci.

[50] Haxby JV, Hoffman EA, Gobbini MI (2002). Human neural systems for face recognition and social communication. Biol Psychiatry..

[51] Redcay E (2008). The superior temporal sulcus performs a common function for social and speech perception: implications for the emergence of autism. Neurosci Biobehav Rev..

[52] Nomi JS, Uddin LQ (2015). Face processing in autism spectrum disorders: from brain regions to brain networks. Neuropsychologia..

[53] Mundy PC (2016). Autism and joint attention: development, neuroscience, and clinical fundamentals.

[54] Morales Michael, Mundy Peter, Delgado Christine E.F., Yale Marygrace, Messinger Daniel, Neal Rebecca, Schwartz Heidi K. (2000). Responding to Joint Attention Across the 6- Through 24-Month Age Period and Early Language Acquisition. Journal of Applied Developmental Psychology.

[55] Mundy P, Block J, Van Hecke AV (2009). Individual differences and the development of joint attention in infancy. Child Dev..

[56] Elison JT, Wolff JJ, Heimer DC, Paterson SJ, Gu H, Hazlett HC (2013). Frontolimbic neural circuitry at 6 months predicts individual differences in joint attention at 9 months. Dev Sci..

[57] Oishi K, Faria AV, Hsu J, Tippett D, Mori S, Hillis AE (2015). Critical role of the right uncinate fasciculus in emotional empathy. Ann Neurol..

[58] Von Der Heide RJ, Skipper LM, Klobusicky E, Olson IR (2013). Dissecting the uncinate fasciculus: disorders, controversies and a hypothesis. Brain..

[59] Wolff JJ, Gu H, Gerig G, Elison JT, Styner M, Gouttard S (2012). Differences in white matter fiber tract development present from 6 to 24 months in infants with autism. Am J Psychiatry..

[60] Ameis SH, Catani M (2015). Altered white matter connectivity as a neural substrate for social impairment in autism spectrum disorder. Cortex..

[61] Kier E, Staib L, Davis L, Bronen R (2004). MR imaging of the temporal stem: anatomic dissection tractography of the uncinate fasciculus, inferior occipitofrontal fasciculus, and Meyer’s loop of the optic radtiation. Am J Neuroradiol..

[62] Bubb EJ, Metzler-Baddeley C, Aggleton JP (2018). The cingulum bundle: anatomy, function, and dysfunction. Neurosci Biobehav Rev..

[63] Pruett JR, Kandala S, Hoertel S, Snyder AZ, Elison JT (2015). Accurate age classification of 6 and 12 month-old infants based on resting-state functional connectivity magnetic resonance imaging data. Dev Cogn Neurosci..

[64] McKinnon CJ, Eggebrecht AT, Todorov A, Wolff JJ, Elison JT, Adams CM (2018). Restricted and repetitive behavior and brain functional connectivity in infants at risk for developing autism spectrum disorder. Biol Psychiatry Cogn Neurosci Neuroimaging..

[65] Vogan VM, Morgan BR, Leung RC, Anagnostou E, Doyle-Thomas K, Taylor MJ (2016). Widespread white matter differences in children and adolescents with autism spectrum disorder. J Autism Dev Disord..

[66] Cheng Y, Chou K-H, Chen I-Y, Fan Y-T, Decety J, Lin C-P (2010). Atypical development of white matter microstructure in adolescents with autism spectrum disorders. Neuroimage..

[67] Maximo JO, Kana RK. Aberrant “deep connectivity” in autism: a cortico-subcortical functional connectivity magnetic resonance imaging study. Autism Res. 2019;(January):116–23. 10.1002/aur.2058 30624021

[68] Wolff JJ, Swanson MR, Elison JT, Gerig G, Pruett JR, Styner MA (2017). Neural circuitry at age 6 months associated with later repetitive behavior and sensory responsiveness in autism. Mol Autism..

[69] O’Reilly C, Lewis JD, Elsabbagh M (2017). Is functional brain connectivity atypical in autism? A systematic review of EEG and MEG studies. PLoS One..

[70] Koldewyn K, Yendiki A, Weigelt S, Gweon H, Julian J, Richardson H (2014). Differences in the right inferior longitudinal fasciculus but no general disruption of white matter tracts in children with autism spectrum disorder. Proc Natl Acad Sci..

[71] Yendiki A, Koldewyn K, Kakunoori S, Kanwisher N, Fischl B (2014). Spurious group differences due to head motion in a diffusion MRI study. Neuroimage..

[72] Vasa RA, Mostofsky SH, Ewen JB (2016). The disrupted connectivity hypothesis of autism spectrum disorders : time for the next phase in research. Biol Psychiatry Cogn Neurosci Neuroimaging..

[73] Solders SK, Carper RA, Müller RA (2017). White matter compromise in autism? Differentiating motion confounds from true differences in diffusion tensor imaging. Autism Res.

[74] Marami B, Scherrer B, Afacan O, Erem B, Warfield SK, Gholipour A (2016). Motion-robust diffusion-weighted brain MRI reconstruction through slice-level registration-based motion tracking. IEEE Trans Med Imaging..

[75] Beaulieu C (2002). The basis of anisotropic water diffusion in the nervous system - a technical review. NMR Biomed..

[76] Scherrer B, Schwartzman A, Taquet M, Prabhu SP, Sahin M, Akhondi-Asl A (2013). Characterizing the distribution of anisotropic micro-structural environments with diffusion-weighted imaging (DIAMOND). Med Image Comput Comput Assist Interv..

