# Supplementary material for: Early white matter development is abnormal in tuberous sclerosis complex patients who develop autism spectrum disorder
Source: J Neurodev Disord. 2019 Dec 16;11:36. doi: 10.1186/s11689-019-9293-x (PMC6912944; doi:10.1186/s11689-019-9293-x)
Supplement: Supplementary file 1 — Additional file 1: Table S1. Clinical T1, T2, and Diffusion-weighted MR protocols 1 for the TACERN study. Table S2. Full longitudinal mixed effect model results for longitudinal trajectories of fractional anisotropy (FA) in each white matter ROI. Table S3. Full longitudinal mixed effect model results for longitudinal trajectories of mean diffusivity (MD) in each white matter ROI. Model estimates are scaled X 1000. [file 11689_2019_9293_MOESM1_ESM.docx]

Table S1. Clinical T1, T2, and Diffusion-weighted MR protocols for the TACERN study.

|  | BCH | | UCLA | CCHMC | | UAB | UTH |
| --- | --- | --- | --- | --- | --- | --- | --- |
| ScannerID | A | B | C | D | E | F | G |
| Field Strength (T) | 3 | 3 | 3 | 3 | 3 | 3 | 3 |
| Manufacturer | Siemens | Siemens | Siemens | Philips | Philips | Philips | General Electric |
| Model | TrioTim | Skyra | TrioTim | Achieva | Ingenia | Ingenia | Signa HDxt |
| Software Versions | syngoMRB17 | syngoMRE11 | syngoMRB17 | 3.2.1 | 5.1.9; 5.3.0 | 4.1.3; 5.1.7; 5.3.0 | HD 16 |
| Number of head coil channels | 32 | 32 | 12 | 32 | 32 | 32 | 8 |
| T1-weighted | |  |  |  |  |  |  |
| Orientation | sagittal | sagittal | sagittal | sagittal | sagittal | sagittal | sagittal |
| Field of view (mm) | 256 x 256 | 224 x 224 | 256 x 256 | 220 x 220 | 220 x 220 | 220 x 220 | 220 x 220 |
| Matrix | 256 x 256 | 256 x 256 | 256 x 256 | 224 x 224 | 224 x 224 | 224 x 224 | 256 x 256 |
| Number of Slices | 176 | 192 | 176 | 176 | 176 | 176 | 172 |
| Resolution (mm) | 1 x 1 x 1 | 0.9 x 0.9 x 0.9 | 1 x 1 x 1 | 1 x 1 x 1 | 1 x 1 x 1 | 1 x 1 x 1 | 0.9 x 0.9 x 1.0 |
| Repetition time (ms) | 8 | 8 | 8 | 8 | 8 | 8 | 6 |
| Echo time (ms) | 4 | 2 | 4 | 4 | 4 | 4 | 3 |
| Bandwidth (Hz/Px) | 199 | 200 | 199 | 191 | 191 | 191 | 244 |
| Inversion Time (ms) | 1100 | 1100 | 1100 | 1100 | 1100 | 1100 | 1100 |
| Flip Angle (deg) | 7 | 7 | 7 | 7 | 7 | 7 | 7 |
| Number of Averages | 1 | 1 | 1 | 1 | 1 | 1 | 1 |
| T2-weighted | |  |  |  |  |  |  |
| Orientation | axial | axial | axial | axial | axial | axial | axial |
| Field of view (mm) | 159 x 200 | 162 x 200 | 159 x 200 | 200 x 200 | 200 x 200 | 200 x 200 | 200 x 200 |
| Matrix | 408 x 512 | 364 x 448 | 408 x 512 | 512 x 512 | 512 x 512 | 512 x 512 | 512 x 512 |
| Number of Slices | 76 | 90 | 76 | 76 | 76 | 76 | 76 |
| Resolution (mm) | 0.4 x 0.4 x 2 | 0.4 x 0.4 x 2 | 0.4 x 0.4 x 2 | 0.4 x 0.4 x 2 | 0.4 x 0.4 x 2 | 0.4 x 0.4 x 2 | 0.4 x 0.4 x 2 |
| Repetition time (ms) | 14850 | 10900 | 14850 | 9366 | 7182 | 10300 | 15000 |
| Echo time (ms) | 79 | 82 | 79 | 79 | 79 | 79 | 76 |
| Bandwidth (Hz/Px) | 208 | 225 | 208 | 196 | 200 | 196 | 244 |
| Flip Angle (deg) | 90 | 90 | 90 | 90 | 90 | 90 | 90 |
| Number of Averages | 2 | 2 | 2 | 2 | 2 | 2 | 2 |
| Diffusion-weighted | |  |  |  |  |  |  |
| Orientation | axial | axial | axial | axial | axial | axial | axial |
| Field of view (mm) | 220 x 220 | 220 x 220 | 220 x 220 | 220 x 220 | 220 x 220 | 220 x 220 | 220 x 220 |
| Matrix | 128 x 128 | 128 x 128 | 128 x 128 | 128 x 128 | 128 x 128 | 128 x 128 | 256 x 256 |
| Number of Slices | 74 | 74 | 74 | 68 | 72 | 72 | 48 |
| Resolution (mm) | 1.7 x 1.7 x 2.0 | 1.7 x 1.7 x 2.0 | 1.7 x 1.7 x 2.0 | 1.7 x 1.7 x 2.0 | 1.7 x 1.7 x 2.0 | 1.7 x 1.7 x 2.0 | 0.9 x 0.9 x 2.0 |
| Repetition time (ms) | 6448 | 6800 | 10900 | 10400 | 11300 | 15000 | 12700 |
| Echo time (ms) | 88 | 94 | 88 | 64 | 98 | 78 | 87 |
| Bandwidth (Hz/Px) | 1395 | 1500 | 1395 | 2378 | 1144 | 1276 | 1953 |
| Flip Angle (deg) | 90 | 90 | 90 | 90 | 90 | 90 | 90 |
| Number of Averages | 1 | 1 | 1 | 1 | 1 | 1 | 1 |
| b-values (number of directions) | 0 (13) | 0 (13) | 0 (15) | 0 (3) | 0 (18) | 0 (24) | 0 (15) |
|  | 400 (6) | 400 (6) | - | - | 400 (6) | - | - |
|  | 600 (6) | 600 (6) | - | - | 600 (6) | - | - |
|  | 800 (6) | 800 (6) | - | - | 800 (6) | - | - |
|  | 1000 (30) | 1000 (30) | 1000 (30) | 1000 (30) | 1000 (30) | 1000 (30) | 1000 (30) |
|  | 1050-1850 (20) | 1050-1850 (20) | 1050-1850 (20) | 1050-1850 (20) | 1050-1850 (20) | 1050-1850 (20) | - |
|  | 2000 (6) | 2000 (6) | 2000 (6) | 2000 (6) | 2000 (6) | 2000 (6) | - |
|  | - | - | 2500 (30) | 2500 (30) | 2500 (30) | 2500 (30) | 2500 (30) |
|  | 3000 (4) | 3000 (4) | 3000 (4) | 3000 (4) | 3000 (4) | 3000 (4) | 3000 (31) |

Table S2. Full longitudinal mixed effect model results for longitudinal trajectories of fractional anisotropy (FA) in each white matter ROI.

| hemi | ROI | Independent Variable | Estimate | Standard Error | t.value | Chisq | Pr(Chi) |
| --- | --- | --- | --- | --- | --- | --- | --- |
| left | arcuate-Broca’s | F23outcome_BIN | -0.0178 | 0.0073 | -2.4470 | 6.07 | 0.0137 |
| left | arcuate-Broca’s | F23outcome_BIN:logbaselineage | -0.0172 | 0.0147 | -1.1646 | 1.40 | 0.2368 |
| left | arcuate-Broca’s | F23outcome_BIN:logyrsfrombaseline | 0.0059 | 0.0060 | 0.9939 | 1.01 | 0.3147 |
| left | arcuate-Broca’s | intercept | 0.3090 | 0.0048 | 64.8020 | - | - |
| left | arcuate-Broca’s | logbaselineage | 0.0479 | 0.0082 | 5.8293 | 30.90 | 0.0000 |
| left | arcuate-Broca’s | logyrsfrombaseline | 0.0463 | 0.0034 | 13.5239 | 102.00 | 0.0000 |
| right | arcuate-Broca’s | F23outcome_BIN | -0.0243 | 0.0072 | -3.3953 | 11.42 | 0.0007 |
| right | arcuate-Broca’s | F23outcome_BIN:logbaselineage | 0.0062 | 0.0145 | 0.4288 | 0.19 | 0.6615 |
| right | arcuate-Broca’s | F23outcome_BIN:logyrsfrombaseline | 0.0063 | 0.0051 | 1.2407 | 1.56 | 0.2116 |
| right | arcuate-Broca’s | intercept | 0.3170 | 0.0047 | 67.6234 | - | - |
| right | arcuate-Broca’s | logbaselineage | 0.0378 | 0.0081 | 4.6620 | 20.59 | 0.0000 |
| right | arcuate-Broca’s | logyrsfrombaseline | 0.0457 | 0.0029 | 15.6006 | 118.83 | 0.0000 |
| left | arcuate-geschwind’s | F23outcome_BIN | -0.0158 | 0.0067 | -2.3529 | 5.62 | 0.0178 |
| left | arcuate-geschwind’s | F23outcome_BIN:logbaselineage | 0.0037 | 0.0136 | 0.2696 | 0.08 | 0.7832 |
| left | arcuate-geschwind’s | F23outcome_BIN:logyrsfrombaseline | 0.0043 | 0.0044 | 0.9670 | 0.96 | 0.3272 |
| left | arcuate-geschwind’s | intercept | 0.2610 | 0.0044 | 59.3878 | - | - |
| left | arcuate-geschwind’s | logbaselineage | 0.0324 | 0.0076 | 4.2610 | 17.47 | 0.0000 |
| left | arcuate-geschwind’s | logyrsfrombaseline | 0.0410 | 0.0025 | 16.1306 | 116.46 | 0.0000 |
| right | arcuate-geschwind’s | F23outcome_BIN | -0.0038 | 0.0060 | -0.6396 | 0.42 | 0.5152 |
| right | arcuate-geschwind’s | F23outcome_BIN:logbaselineage | 0.0127 | 0.0122 | 1.0415 | 1.12 | 0.2896 |
| right | arcuate-geschwind’s | F23outcome_BIN:logyrsfrombaseline | -0.0005 | 0.0040 | -0.1226 | 0.02 | 0.9024 |
| right | arcuate-geschwind’s | intercept | 0.2605 | 0.0039 | 66.1657 | - | - |
| right | arcuate-geschwind’s | logbaselineage | 0.0343 | 0.0068 | 5.0318 | 23.64 | 0.0000 |
| right | arcuate-geschwind’s | logyrsfrombaseline | 0.0393 | 0.0023 | 17.0847 | 124.41 | 0.0000 |
| left | arcuate-Wernicke’s | F23outcome_BIN | -0.0227 | 0.0064 | -3.5504 | 12.41 | 0.0004 |
| left | arcuate-Wernicke’s | F23outcome_BIN:logbaselineage | -0.0063 | 0.0130 | -0.4845 | 0.24 | 0.6216 |
| left | arcuate-Wernicke’s | F23outcome_BIN:logyrsfrombaseline | 0.0051 | 0.0049 | 1.0388 | 1.10 | 0.2948 |
| left | arcuate-Wernicke’s | intercept | 0.2642 | 0.0042 | 63.1212 | - | - |
| left | arcuate-Wernicke’s | logbaselineage | 0.0352 | 0.0072 | 4.8717 | 22.28 | 0.0000 |
| left | arcuate-Wernicke’s | logyrsfrombaseline | 0.0363 | 0.0028 | 12.8227 | 91.89 | 0.0000 |
| right | arcuate-Wernicke’s | F23outcome_BIN | -0.0133 | 0.0068 | -1.9716 | 3.97 | 0.0463 |
| right | arcuate-Wernicke’s | F23outcome_BIN:logbaselineage | 0.0037 | 0.0137 | 0.2689 | 0.08 | 0.7837 |
| right | arcuate-Wernicke’s | F23outcome_BIN:logyrsfrombaseline | 0.0023 | 0.0046 | 0.5043 | 0.26 | 0.6095 |
| right | arcuate-Wernicke’s | intercept | 0.2581 | 0.0044 | 58.3248 | - | - |
| right | arcuate-Wernicke’s | logbaselineage | 0.0280 | 0.0077 | 3.6585 | 13.15 | 0.0003 |
| right | arcuate-Wernicke’s | logyrsfrombaseline | 0.0320 | 0.0027 | 12.0217 | 84.33 | 0.0000 |
| right | anterior limb internal capsule | F23outcome_BIN | -0.0195 | 0.0064 | -3.0365 | 9.22 | 0.0024 |
| right | anterior limb internal capsule | F23outcome_BIN:logbaselineage | 0.0009 | 0.0130 | 0.0699 | 0.01 | 0.9425 |
| right | anterior limb internal capsule | F23outcome_BIN:logyrsfrombaseline | 0.0029 | 0.0049 | 0.5769 | 0.34 | 0.5587 |
| right | anterior limb internal capsule | intercept | 0.3760 | 0.0042 | 89.3011 | - | - |
| right | anterior limb internal capsule | logbaselineage | 0.0448 | 0.0073 | 6.1605 | 34.04 | 0.0000 |
| right | anterior limb internal capsule | logyrsfrombaseline | 0.0345 | 0.0028 | 12.1569 | 87.50 | 0.0000 |
| left | anterior limb internal capsule | F23outcome_BIN | -0.0145 | 0.0070 | -2.0828 | 4.42 | 0.0355 |
| left | anterior limb internal capsule | F23outcome_BIN:logbaselineage | 0.0096 | 0.0141 | 0.6764 | 0.47 | 0.4911 |
| left | anterior limb internal capsule | F23outcome_BIN:logyrsfrombaseline | 0.0024 | 0.0052 | 0.4534 | 0.21 | 0.6464 |
| left | anterior limb internal capsule | intercept | 0.3487 | 0.0046 | 76.3927 | - | - |
| left | anterior limb internal capsule | logbaselineage | 0.0394 | 0.0079 | 5.0010 | 23.53 | 0.0000 |
| left | anterior limb internal capsule | logyrsfrombaseline | 0.0365 | 0.0030 | 12.1865 | 87.48 | 0.0000 |
| midline | corpus callosum | F23outcome_BIN | -0.0236 | 0.0069 | -3.4320 | 11.66 | 0.0006 |
| midline | corpus callosum | F23outcome_BIN:logbaselineage | -0.0085 | 0.0139 | -0.6098 | 0.39 | 0.5347 |
| midline | corpus callosum | F23outcome_BIN:logyrsfrombaseline | 0.0069 | 0.0054 | 1.2689 | 1.63 | 0.2012 |
| midline | corpus callosum | intercept | 0.5293 | 0.0045 | 117.9020 | - | - |
| midline | corpus callosum | logbaselineage | 0.0655 | 0.0077 | 8.4545 | 57.21 | 0.0000 |
| midline | corpus callosum | logyrsfrombaseline | 0.0615 | 0.0031 | 19.6491 | 152.61 | 0.0000 |
| left | cingulum | F23outcome_BIN | -0.0111 | 0.0053 | -2.0958 | 4.48 | 0.0343 |
| left | cingulum | F23outcome_BIN:logbaselineage | -0.0086 | 0.0107 | -0.7989 | 0.66 | 0.4164 |
| left | cingulum | F23outcome_BIN:logyrsfrombaseline | 0.0020 | 0.0039 | 0.5094 | 0.27 | 0.6052 |
| left | cingulum | intercept | 0.2949 | 0.0035 | 85.0387 | - | - |
| left | cingulum | logbaselineage | 0.0370 | 0.0060 | 6.1778 | 34.00 | 0.0000 |
| left | cingulum | logyrsfrombaseline | 0.0358 | 0.0022 | 16.1747 | 120.46 | 0.0000 |
| right | cingulum | F23outcome_BIN | -0.0098 | 0.0050 | -1.9624 | 3.94 | 0.0473 |
| right | cingulum | F23outcome_BIN:logbaselineage | 0.0001 | 0.0101 | 0.0070 | 0.00 | 0.9936 |
| right | cingulum | F23outcome_BIN:logyrsfrombaseline | 0.0068 | 0.0039 | 1.7347 | 3.05 | 0.0808 |
| right | cingulum | intercept | 0.2872 | 0.0033 | 88.1570 | - | - |
| right | cingulum | logbaselineage | 0.0336 | 0.0056 | 5.9829 | 32.17 | 0.0000 |
| right | cingulum | logyrsfrombaseline | 0.0292 | 0.0022 | 12.9745 | 92.53 | 0.0000 |
| right | inferior extreme capsule | F23outcome_BIN | -0.0020 | 0.0052 | -0.3891 | 0.16 | 0.6920 |
| right | inferior extreme capsule | F23outcome_BIN:logbaselineage | 0.0100 | 0.0106 | 0.9430 | 0.92 | 0.3375 |
| right | inferior extreme capsule | F23outcome_BIN:logyrsfrombaseline | -0.0023 | 0.0033 | -0.6949 | 0.49 | 0.4837 |
| right | inferior extreme capsule | intercept | 0.2726 | 0.0034 | 79.7596 | - | - |
| right | inferior extreme capsule | logbaselineage | 0.0289 | 0.0059 | 4.8805 | 22.38 | 0.0000 |
| right | inferior extreme capsule | logyrsfrombaseline | 0.0225 | 0.0019 | 11.7976 | 79.37 | 0.0000 |
| left | inferior extreme capsule | F23outcome_BIN | -0.0059 | 0.0055 | -1.0681 | 1.18 | 0.2776 |
| left | inferior extreme capsule | F23outcome_BIN:logbaselineage | -0.0017 | 0.0112 | -0.1540 | 0.02 | 0.8752 |
| left | inferior extreme capsule | F23outcome_BIN:logyrsfrombaseline | 0.0007 | 0.0042 | 0.1679 | 0.03 | 0.8631 |
| left | inferior extreme capsule | intercept | 0.2708 | 0.0036 | 74.8193 | - | - |
| left | inferior extreme capsule | logbaselineage | 0.0275 | 0.0063 | 4.4045 | 18.57 | 0.0000 |
| left | inferior extreme capsule | logyrsfrombaseline | 0.0207 | 0.0024 | 8.4788 | 50.06 | 0.0000 |
| right | Posterior limb internal capsule | F23outcome_BIN | -0.0032 | 0.0059 | -0.5481 | 0.31 | 0.5760 |
| right | Posterior limb internal capsule | F23outcome_BIN:logbaselineage | 0.0028 | 0.0118 | 0.2407 | 0.06 | 0.8076 |
| right | Posterior limb internal capsule | F23outcome_BIN:logyrsfrombaseline | -0.0022 | 0.0061 | -0.3539 | 0.13 | 0.7215 |
| right | Posterior limb internal capsule | intercept | 0.4887 | 0.0038 | 127.1897 | - | - |
| right | Posterior limb internal capsule | logbaselineage | 0.0388 | 0.0066 | 5.9075 | 31.48 | 0.0000 |
| right | Posterior limb internal capsule | logyrsfrombaseline | 0.0325 | 0.0035 | 9.2411 | 56.88 | 0.0000 |
| left | Posterior limb internal capsule | F23outcome_BIN | -0.0086 | 0.0072 | -1.1907 | 1.46 | 0.2262 |
| left | Posterior limb internal capsule | F23outcome_BIN:logbaselineage | -0.0052 | 0.0144 | -0.3644 | 0.14 | 0.7095 |
| left | Posterior limb internal capsule | F23outcome_BIN:logyrsfrombaseline | -0.0057 | 0.0079 | -0.7195 | 0.53 | 0.4681 |
| left | Posterior limb internal capsule | intercept | 0.5006 | 0.0047 | 106.7590 | - | - |
| left | Posterior limb internal capsule | logbaselineage | 0.0455 | 0.0080 | 5.6936 | 29.59 | 0.0000 |
| left | Posterior limb internal capsule | logyrsfrombaseline | 0.0368 | 0.0046 | 8.0509 | 45.80 | 0.0000 |
| right | Sagittal stratum | F23outcome_BIN | -0.0225 | 0.0088 | -2.5424 | 6.55 | 0.0105 |
| right | Sagittal stratum | F23outcome_BIN:logbaselineage | 0.0233 | 0.0177 | 1.3124 | 1.78 | 0.1827 |
| right | Sagittal stratum | F23outcome_BIN:logyrsfrombaseline | 0.0349 | 0.0093 | 3.7441 | 13.48 | 0.0002 |
| right | Sagittal stratum | intercept | 0.4070 | 0.0058 | 70.4950 | - | - |
| right | Sagittal stratum | logbaselineage | 0.0176 | 0.0099 | 1.7792 | 3.25 | 0.0716 |
| right | Sagittal stratum | logyrsfrombaseline | 0.0143 | 0.0054 | 2.6684 | 6.98 | 0.0082 |
| left | Sagittal stratum | F23outcome_BIN | -0.0029 | 0.0095 | -0.3086 | 0.10 | 0.7539 |
| left | Sagittal stratum | F23outcome_BIN:logbaselineage | 0.0227 | 0.0191 | 1.1861 | 1.45 | 0.2282 |
| left | Sagittal stratum | F23outcome_BIN:logyrsfrombaseline | 0.0038 | 0.0083 | 0.4602 | 0.22 | 0.6399 |
| left | Sagittal stratum | intercept | 0.3912 | 0.0062 | 63.1593 | - | - |
| left | Sagittal stratum | logbaselineage | 0.0232 | 0.0107 | 2.1782 | 4.83 | 0.0279 |
| left | Sagittal stratum | logyrsfrombaseline | 0.0269 | 0.0048 | 5.6369 | 27.69 | 0.0000 |

Table S3. Full longitudinal mixed effect model results for longitudinal trajectories of mean diffusivity (MD) in each white matter ROI. Model estimates are scaled X 1000.

| hemi | ROI | Independent Variable | Estimate | Standard Error | t.value | Chisq | Pr(Chi) |
| --- | --- | --- | --- | --- | --- | --- | --- |
| left | arcuate-Broca’s | F23outcome_BIN | 0.0108 | 0.0131 | 0.8294 | 0.71 | 0.3995 |
| left | arcuate-Broca’s | F23outcome_BIN:logbaselineage | 0.0229 | 0.0263 | 0.8722 | 0.78 | 0.3758 |
| left | arcuate-Broca’s | F23outcome_BIN:logyrsfrombaseline | -0.0040 | 0.0123 | -0.3280 | 0.11 | 0.7368 |
| left | arcuate-Broca’s | intercept | 0.9408 | 0.0085 | 110.2001 | - | - |
| left | arcuate-Broca’s | logbaselineage | -0.0790 | 0.0147 | -5.3904 | 26.84 | 0.0000 |
| left | arcuate-Broca’s | logyrsfrombaseline | -0.0943 | 0.0071 | -13.3513 | 95.40 | 0.0000 |
| right | arcuate-Broca’s | F23outcome_BIN | 0.0249 | 0.0129 | 1.9331 | 3.82 | 0.0505 |
| right | arcuate-Broca’s | F23outcome_BIN:logbaselineage | 0.0022 | 0.0260 | 0.0835 | 0.01 | 0.9333 |
| right | arcuate-Broca’s | F23outcome_BIN:logyrsfrombaseline | -0.0178 | 0.0112 | -1.5941 | 2.58 | 0.1080 |
| right | arcuate-Broca’s | intercept | 0.9496 | 0.0084 | 112.8721 | - | - |
| right | arcuate-Broca’s | logbaselineage | -0.0631 | 0.0145 | -4.3572 | 18.14 | 0.0000 |
| right | arcuate-Broca’s | logyrsfrombaseline | -0.0807 | 0.0064 | -12.5758 | 92.40 | 0.0000 |
| left | arcuate-geschwind’s | F23outcome_BIN | 0.0286 | 0.0167 | 1.7117 | 3.01 | 0.0830 |
| left | arcuate-geschwind’s | F23outcome_BIN:logbaselineage | 0.0518 | 0.0339 | 1.5291 | 2.40 | 0.1210 |
| left | arcuate-geschwind’s | F23outcome_BIN:logyrsfrombaseline | -0.0133 | 0.0112 | -1.1861 | 1.43 | 0.2314 |
| left | arcuate-geschwind’s | intercept | 0.9995 | 0.0109 | 91.4666 | - | - |
| left | arcuate-geschwind’s | logbaselineage | -0.0907 | 0.0189 | -4.7971 | 21.70 | 0.0000 |
| left | arcuate-geschwind’s | logyrsfrombaseline | -0.0946 | 0.0064 | -14.7513 | 109.44 | 0.0000 |
| right | arcuate-geschwind’s | F23outcome_BIN | 0.0076 | 0.0160 | 0.4787 | 0.24 | 0.6264 |
| right | arcuate-geschwind’s | F23outcome_BIN:logbaselineage | 0.0122 | 0.0324 | 0.3774 | 0.15 | 0.7011 |
| right | arcuate-geschwind’s | F23outcome_BIN:logyrsfrombaseline | 0.0022 | 0.0112 | 0.1931 | 0.04 | 0.8450 |
| right | arcuate-geschwind’s | intercept | 1.0136 | 0.0105 | 96.9500 | - | - |
| right | arcuate-geschwind’s | logbaselineage | -0.0860 | 0.0181 | -4.7551 | 21.37 | 0.0000 |
| right | arcuate-geschwind’s | logyrsfrombaseline | -0.0977 | 0.0064 | -15.2236 | 112.75 | 0.0000 |
| left | arcuate-Wernicke’s | F23outcome_BIN | 0.0053 | 0.0163 | 0.3220 | 0.11 | 0.7431 |
| left | arcuate-Wernicke’s | F23outcome_BIN:logbaselineage | 0.0151 | 0.0332 | 0.4558 | 0.22 | 0.6426 |
| left | arcuate-Wernicke’s | F23outcome_BIN:logyrsfrombaseline | -0.0039 | 0.0095 | -0.4099 | 0.17 | 0.6779 |
| left | arcuate-Wernicke’s | intercept | 0.9798 | 0.0107 | 91.7341 | - | - |
| left | arcuate-Wernicke’s | logbaselineage | -0.0519 | 0.0185 | -2.8011 | 7.86 | 0.0050 |
| left | arcuate-Wernicke’s | logyrsfrombaseline | -0.0741 | 0.0054 | -13.6370 | 98.68 | 0.0000 |
| right | arcuate-Wernicke’s | F23outcome_BIN | 0.0060 | 0.0146 | 0.4114 | 0.18 | 0.6754 |
| right | arcuate-Wernicke’s | F23outcome_BIN:logbaselineage | 0.0227 | 0.0296 | 0.7690 | 0.61 | 0.4340 |
| right | arcuate-Wernicke’s | F23outcome_BIN:logyrsfrombaseline | -0.0026 | 0.0094 | -0.2778 | 0.08 | 0.7768 |
| right | arcuate-Wernicke’s | intercept | 0.9755 | 0.0095 | 102.3542 | - | - |
| right | arcuate-Wernicke’s | logbaselineage | -0.0657 | 0.0165 | -3.9826 | 15.36 | 0.0001 |
| right | arcuate-Wernicke’s | logyrsfrombaseline | -0.0705 | 0.0054 | -13.0803 | 93.16 | 0.0000 |
| right | anterior limb internal capsule | F23outcome_BIN | -0.0088 | 0.0087 | -1.0184 | 1.07 | 0.3001 |
| right | anterior limb internal capsule | F23outcome_BIN:logbaselineage | 0.0165 | 0.0174 | 0.9485 | 0.93 | 0.3352 |
| right | anterior limb internal capsule | F23outcome_BIN:logyrsfrombaseline | 0.0058 | 0.0085 | 0.6793 | 0.47 | 0.4944 |
| right | anterior limb internal capsule | intercept | 0.8921 | 0.0057 | 157.4847 | - | - |
| right | anterior limb internal capsule | logbaselineage | -0.0612 | 0.0097 | -6.2976 | 35.18 | 0.0000 |
| right | anterior limb internal capsule | logyrsfrombaseline | -0.0543 | 0.0049 | -11.1287 | 77.08 | 0.0000 |
| left | anterior limb internal capsule | F23outcome_BIN | -0.0094 | 0.0114 | -0.8219 | 0.70 | 0.4027 |
| left | anterior limb internal capsule | F23outcome_BIN:logbaselineage | 0.0243 | 0.0231 | 1.0492 | 1.14 | 0.2863 |
| left | anterior limb internal capsule | F23outcome_BIN:logyrsfrombaseline | 0.0074 | 0.0089 | 0.8352 | 0.71 | 0.3997 |
| left | anterior limb internal capsule | intercept | 0.8926 | 0.0075 | 119.4258 | - | - |
| left | anterior limb internal capsule | logbaselineage | -0.0556 | 0.0129 | -4.3104 | 17.88 | 0.0000 |
| left | anterior limb internal capsule | logyrsfrombaseline | -0.0566 | 0.0051 | -11.1158 | 76.17 | 0.0000 |
| midline | corpus callosum | F23outcome_BIN | 0.0280 | 0.0164 | 1.7016 | 2.97 | 0.0848 |
| midline | corpus callosum | F23outcome_BIN:logbaselineage | 0.0492 | 0.0333 | 1.4776 | 2.25 | 0.1339 |
| midline | corpus callosum | F23outcome_BIN:logyrsfrombaseline | -0.0019 | 0.0129 | -0.1474 | 0.02 | 0.8790 |
| midline | corpus callosum | intercept | 1.1204 | 0.0108 | 104.1608 | - | - |
| midline | corpus callosum | logbaselineage | -0.1394 | 0.0186 | -7.5095 | 47.26 | 0.0000 |
| midline | corpus callosum | logyrsfrombaseline | -0.1218 | 0.0074 | -16.4308 | 122.29 | 0.0000 |
| left | cingulum | F23outcome_BIN | -0.0054 | 0.0081 | -0.6657 | 0.46 | 0.4969 |
| left | cingulum | F23outcome_BIN:logbaselineage | 0.0249 | 0.0162 | 1.5306 | 2.41 | 0.1206 |
| left | cingulum | F23outcome_BIN:logyrsfrombaseline | 0.0018 | 0.0089 | 0.2004 | 0.04 | 0.8388 |
| left | cingulum | intercept | 0.9637 | 0.0053 | 181.7612 | - | - |
| left | cingulum | logbaselineage | -0.0793 | 0.0090 | -8.7679 | 61.02 | 0.0000 |
| left | cingulum | logyrsfrombaseline | -0.0847 | 0.0051 | -16.4532 | 130.31 | 0.0000 |
| right | cingulum | F23outcome_BIN | -0.0069 | 0.0078 | -0.8819 | 0.81 | 0.3690 |
| right | cingulum | F23outcome_BIN:logbaselineage | 0.0175 | 0.0156 | 1.1217 | 1.30 | 0.2546 |
| right | cingulum | F23outcome_BIN:logyrsfrombaseline | -0.0039 | 0.0085 | -0.4629 | 0.22 | 0.6406 |
| right | cingulum | intercept | 0.9658 | 0.0051 | 189.5402 | - | - |
| right | cingulum | logbaselineage | -0.0785 | 0.0087 | -9.0199 | 63.76 | 0.0000 |
| right | cingulum | logyrsfrombaseline | -0.0791 | 0.0049 | -16.1571 | 129.13 | 0.0000 |
| right | inferior extreme capsule | F23outcome_BIN | -0.0056 | 0.0067 | -0.8349 | 0.72 | 0.3945 |
| right | inferior extreme capsule | F23outcome_BIN:logbaselineage | 0.0109 | 0.0134 | 0.8151 | 0.68 | 0.4079 |
| right | inferior extreme capsule | F23outcome_BIN:logyrsfrombaseline | 0.0116 | 0.0078 | 1.4826 | 2.22 | 0.1365 |
| right | inferior extreme capsule | intercept | 0.9473 | 0.0044 | 215.4673 | - | - |
| right | inferior extreme capsule | logbaselineage | -0.0649 | 0.0075 | -8.6764 | 59.54 | 0.0000 |
| right | inferior extreme capsule | logyrsfrombaseline | -0.0579 | 0.0045 | -12.8362 | 95.32 | 0.0000 |
| left | inferior extreme capsule | F23outcome_BIN | -0.0047 | 0.0077 | -0.6115 | 0.39 | 0.5325 |
| left | inferior extreme capsule | F23outcome_BIN:logbaselineage | 0.0197 | 0.0155 | 1.2720 | 1.67 | 0.1966 |
| left | inferior extreme capsule | F23outcome_BIN:logyrsfrombaseline | 0.0097 | 0.0085 | 1.1451 | 1.34 | 0.2476 |
| left | inferior extreme capsule | intercept | 0.9415 | 0.0050 | 186.5361 | - | - |
| left | inferior extreme capsule | logbaselineage | -0.0655 | 0.0086 | -7.6047 | 48.95 | 0.0000 |
| left | inferior extreme capsule | logyrsfrombaseline | -0.0586 | 0.0049 | -11.9861 | 87.95 | 0.0000 |
| right | Posterior limb internal capsule | F23outcome_BIN | -0.0040 | 0.0086 | -0.4726 | 0.23 | 0.6295 |
| right | Posterior limb internal capsule | F23outcome_BIN:logbaselineage | 0.0168 | 0.0173 | 0.9747 | 0.98 | 0.3220 |
| right | Posterior limb internal capsule | F23outcome_BIN:logyrsfrombaseline | 0.0063 | 0.0073 | 0.8516 | 0.74 | 0.3908 |
| right | Posterior limb internal capsule | intercept | 0.8471 | 0.0056 | 151.4999 | - | - |
| right | Posterior limb internal capsule | logbaselineage | -0.0447 | 0.0096 | -4.6420 | 20.43 | 0.0000 |
| right | Posterior limb internal capsule | logyrsfrombaseline | -0.0413 | 0.0042 | -9.7769 | 65.29 | 0.0000 |
| left | Posterior limb internal capsule | F23outcome_BIN | -0.0058 | 0.0104 | -0.5553 | 0.32 | 0.5711 |
| left | Posterior limb internal capsule | F23outcome_BIN:logbaselineage | 0.0248 | 0.0211 | 1.1756 | 1.43 | 0.2324 |
| left | Posterior limb internal capsule | F23outcome_BIN:logyrsfrombaseline | 0.0037 | 0.0088 | 0.4233 | 0.18 | 0.6699 |
| left | Posterior limb internal capsule | intercept | 0.8350 | 0.0068 | 122.4819 | - | - |
| left | Posterior limb internal capsule | logbaselineage | -0.0403 | 0.0117 | -3.4293 | 11.61 | 0.0007 |
| left | Posterior limb internal capsule | logyrsfrombaseline | -0.0452 | 0.0051 | -8.9132 | 54.97 | 0.0000 |
| right | Sagittal stratum | F23outcome_BIN | 0.0216 | 0.0234 | 0.9241 | 0.88 | 0.3477 |
| right | Sagittal stratum | F23outcome_BIN:logbaselineage | 0.0452 | 0.0466 | 0.9717 | 0.97 | 0.3237 |
| right | Sagittal stratum | F23outcome_BIN:logyrsfrombaseline | -0.0555 | 0.0273 | -2.0366 | 4.17 | 0.0411 |
| right | Sagittal stratum | intercept | 1.0895 | 0.0152 | 71.4721 | - | - |
| right | Sagittal stratum | logbaselineage | -0.0677 | 0.0259 | -2.6115 | 6.90 | 0.0086 |
| right | Sagittal stratum | logyrsfrombaseline | -0.0328 | 0.0157 | -2.0847 | 4.34 | 0.0372 |
| left | Sagittal stratum | F23outcome_BIN | 0.0040 | 0.0219 | 0.1819 | 0.03 | 0.8537 |
| left | Sagittal stratum | F23outcome_BIN:logbaselineage | 0.0189 | 0.0445 | 0.4254 | 0.19 | 0.6655 |
| left | Sagittal stratum | F23outcome_BIN:logyrsfrombaseline | 0.0212 | 0.0166 | 1.2746 | 1.64 | 0.1999 |
| left | Sagittal stratum | intercept | 1.1072 | 0.0144 | 77.1090 | - | - |
| left | Sagittal stratum | logbaselineage | -0.0463 | 0.0248 | -1.8678 | 3.57 | 0.0588 |
| left | Sagittal stratum | logyrsfrombaseline | -0.0584 | 0.0095 | -6.1287 | 32.07 | 0.0000 |
